# Microbial Communities Under Distinct Thermal and Geochemical Regimes in Axial and Off-Axis Sediments of Guaymas Basin

**DOI:** 10.3389/fmicb.2021.633649

**Published:** 2021-02-12

**Authors:** Andreas Teske, Gunter Wegener, Jeffrey P. Chanton, Dylan White, Barbara MacGregor, Daniel Hoer, Dirk de Beer, Guangchao Zhuang, Matthew A. Saxton, Samantha B. Joye, Daniel Lizarralde, S. Adam Soule, S. Emil Ruff

**Affiliations:** ^1^Department of Marine Sciences, University of North Carolina at Chapel Hill, Chapel Hill, NC, United States; ^2^Max-Planck-Institute for Marine Microbiology, Bremen, Germany; ^3^MARUM, Center for Marine Environmental Sciences, University of Bremen, Bremen, Germany; ^4^Department of Earth, Ocean and Atmospheric Sciences, Florida State University, Tallahassee, FL, United States; ^5^Department of Earth and Environmental Sciences, University of Minnesota, St. Paul, MI, United States; ^6^Department of Organismic and Evolutionary Biology, Harvard University, Cambridge, MA, United States; ^7^United States Environmental Protection Agency, Research Triangle Park, NC, United States; ^8^Frontiers Science Centre for Deep Ocean Multispheres and Earth System (FDOMES)/Key Laboratory of Marine Chemistry Theory and Technology, Ministry of Education, Ocean University of China, Qingdao, China; ^9^Laboratory for Marine Ecology and Environmental Science, Qingdao National Laboratory for Marine Science and Technology, Ocean University of China, Qingdao, China; ^10^Department of Marine Sciences, University of Georgia, Athens, GA, United States; ^11^Department of Biological Sciences, Miami University, Oxford, OH, United States; ^12^Geology & Geophysics Department, Woods Hole Oceanographic Institution, Woods Hole, MA, United States; ^13^Marine Biological Laboratory, The Ecosystems Center, Woods Hole, MA, United States; ^14^Marine Biological Laboratory, The Josephine Bay Paul Center for Comparative Molecular Biology and Evolution, Woods Hole, MA, United States

**Keywords:** cold seep, hydrothermal sediment, porewater profiles, bacteria, archaea, Guaymas Basin

## Abstract

Cold seeps and hydrothermal vents are seafloor habitats fueled by subsurface energy sources. Both habitat types coexist in Guaymas Basin in the Gulf of California, providing an opportunity to compare microbial communities with distinct physiologies adapted to different thermal regimes. Hydrothermally active sites in the southern Guaymas Basin axial valley, and cold seep sites at Octopus Mound, a carbonate mound with abundant methanotrophic cold seep fauna at the Central Seep location on the northern off-axis flanking regions, show consistent geochemical and microbial differences between hot, temperate, cold seep, and background sites. The changing microbial actors include autotrophic and heterotrophic bacterial and archaeal lineages that catalyze sulfur, nitrogen, and methane cycling, organic matter degradation, and hydrocarbon oxidation. Thermal, biogeochemical, and microbiological characteristics of the sampling locations indicate that sediment thermal regime and seep-derived or hydrothermal energy sources structure the microbial communities at the sediment surface.

## Introduction

Globally, over 700 marine hydrothermal vent sites are currently known (Beaulieu and Szafranski, 2020), including sedimented hydrothermal systems at coastal and continental margin locations (Price and Giovanelli, 2017). One of the best-studied sedimented hydrothermal systems is Guaymas Basin in the Gulf of California, a young marginal rift basin characterized by active seafloor spreading and rapid deposition of organic-rich sediments from highly productive overlying waters (Lonsdale and Becker, 1985). Buried organic matter is hydrothermally transformed to methane, aliphatic and aromatic hydrocarbons, dissolved inorganic carbon, and ammonia, resulting in well-buffered and nutrient-rich vent fluids (Von Damm et al., 1985) that sustain ample microbial communities. The microbiology and biogeochemistry of hydrothermal sediments in Guaymas Basin have been studied extensively. Sulfur-oxidizing microbial mats occur in visually conspicuous hot spots where sulfide, methane-, and hydrocarbon-rich hydrothermal fluids rise to the sediment surface (McKay et al., 2012; MacGregor et al., 2013). Surficial sediments harbor complex anaerobic microbial communities adapted to these conditions, including thermophilic methane- and alkane-oxidizing archaea (Teske et al., 2002; Biddle et al., 2012; Wegener et al., 2015; Dowell et al., 2016; Laso-Pérez et al., 2016; Wang et al., 2019) and hydrocarbon-oxidizing, free-living or syntrophic sulfate-reducing bacteria (reviewed by Teske, 2019). The diversity of hydrothermal regimes in Guaymas Basin selects for microbial communities with adaptations to different thermal and geochemical niches (McKay et al., 2016; Teske et al., 2016). Here we compare thermal gradients, porewater geochemistry, and microbial community composition in the surface layer of different hydrothermal, cold seep, and background sediments (Table 1) to determine whether sediments with distinct porewater geochemistry and thermal regimes harbor particular bacterial and archaeal populations.

**Table 1:** Sampling sites and *Alvin* core numbers for 2016 sediment samples collected during Atlantis cruise AT37-06, used in parallel molecular and biogeochemical analyses.

Guaymas Basin area	Sampling location	Date	Latitude/longitude	Water depth (m)	*Alvin* core number for Sequence data	*Alvin* core number for Porewater data	Sample type	Microbial mat and/or invertebrates	T max.
Southern axial valley	Mat Mound Massif	December 12, 2016	27°00.44'/111°24.52'	2,002	4861-26	no data	Hot hydrothermal vent	White sulfur mat, snails	53°C
Southern axial valley	Mat Mound Massif	December 13, 2016	27°00.43'/111°24.56'	2,000	4862-08	4862-08	Hot hydrothermal vent	Orange *Beggiatoaceae* mat	146°C
Southern axial valley	Mat Mound Massif	December 13, 2016	27°00.43'/111°24.56'	2,000	4862-33	4862-33	Background sediment	Cold bare sediment	3.4°C
Central seep	Octopus Mound Periphery	December 18, 2016	27°28.17'/111°28.39'	1,832	4867-08	4867-32	Background sediment	Cold bare sediment	3°C
Central seep	Octopus Mound Active site	December 18, 2016	27°28.17'/111°28.39'	1,832	4867-14	4867-30	Cold seep	Tubeworms, clams, ampharetid worms	3°C
Southern axial valley	Mat Mound Massif	December 21, 2016	27°00.45'/111°24.54'	2,001	4869-25	4869-26	Hot hydrothermal vent	Orange *Beggiatoaceae* mat	85°C
Southern axial valley	Cathedral Hill	December 22, 2016	27°00.71'/111°24.22'	2,009	4870-16	4870-29	Hot hydrothermal vent	Oily sediment and fluffy white mat	80°C
Southern axial valley	Aceto Balsamico	December 22, 2016	27°00.47'/111°24.43'	2,007	4870-02	4870-32	Temperate hydrothermal vent	Yellow sulfur mat	29°C
Southern axial valley	Northern Towers	December 23, 2016	27°02.77'/111°23.09'	1,994	4871-20	4871-20	Temperate hydrothermal vent	White sulfur mat, worms	13°C
Southern axial valley	Northern Towers	December 23, 2016	27°02.75'/111°23.05'	1,990	4871-26	4871-23	Hot hydrothermal vent	White sulfur mat, no worms	92°C

Thermal gradients are tabulated in Supplementary Tables S2 and S3, and plotted in Supplementary Figure S3. The samples are sorted by *Alvin* dive number.

**Figure 1:**
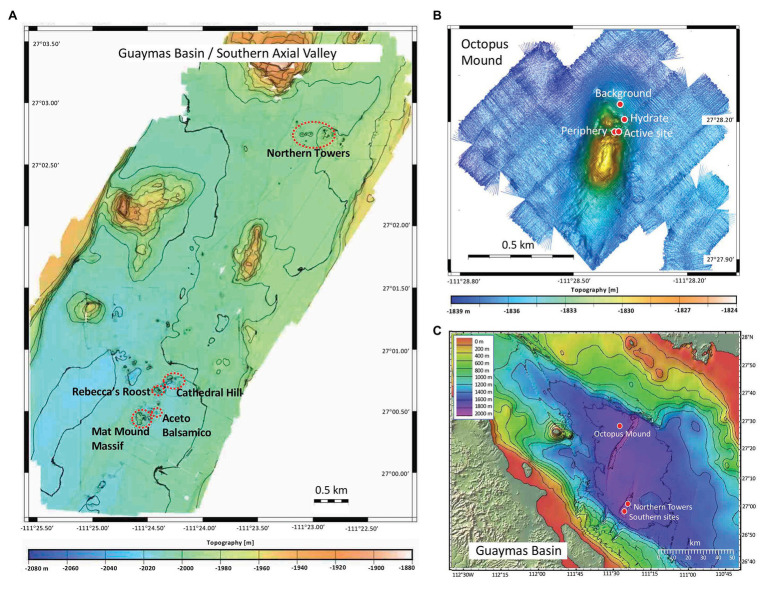
Bathymetric maps of Guaymas Basin and Expedition AT37-06 sampling locations. **(A)** Guaymas Basin southern axial valley, mapped during *Sentry* dives 407-409 and 413-417. **(B)** Central Seep area with Octopus Mound, mapped during *Sentry* dive 412. **(C)** Guaymas Basin overview annotated with sampling sites, based on a template courtesy of C. Mortera, UNAM.

In contrast to previous small-scale surveys that focused on individual hydrothermal mounds or microbial mats (McKay et al., 2012, 2016; Dowell et al., 2016), the sampling sites are separated by distances of hundreds of meters or several miles (Figure 1). Within a few miles of each other, the greater Guaymas Basin geo-ecosystem includes hydrothermal areas at the southern Guaymas Basin spreading center (Teske et al., 2016), off-axis hydrothermal sites (Teske et al., 2019; Ramírez et al., 2020), hydrothermal mounds just off the northern spreading center (Berndt et al., 2016), and cold seeps on the northern flanking regions and the adjacent Sonora Margin (Geilert et al., 2018). Methane-rich off-axis cold seeps have been documented by deep-tow photography of faunal communities and carbonates, and by *in situ* measurements of methane anomalies in the deep water column (Lizarralde et al., 2011), and some cold seep sites have been sampled further by multicoring and gravity coring of sediments and dredging of seafloor minerals (Geilert et al., 2018; Núñez-Useche et al., 2018). In contrast to compression-induced seepage on massively sedimented continental margins and plate boundaries (Suess, 2010), seismic surveys have linked the Guaymas Basin cold seeps to deeply buried volcanic sills that extend from the spreading centers (Einsele et al., 1980) up to 50km across the sedimented Guaymas flanking regions (Lizarralde et al., 2011). At specific off-axis locations, shallow hot sills drive hydrothermal fluid and gas circulation (Teske et al., 2019). Yet in most cases, off-axis sills and their seep fluids have cooled off over time, and methane-rich fluids have temperatures near ambient bottom water when they are released at the sediment surface (Lizarralde et al., 2011; Geilert et al., 2018). Guaymas Basin cold seep sites remain to be explored and sampled up close by ROV and submersible. This study concludes by surveying a cold seep site (“Central Seep”) approximately equidistant from Sonora and Baja California on the northern flanking regions (Geilert et al., 2018; Núñez-Useche et al., 2018).

## Materials and Methods

### Field Survey and Sampling

Guaymas Basin sites were visited and sampled with R/V *Atlantis*, HOV *Alvin*, and AUV *Sentry* during cruise AT37-06 (December 6–29, 2016). *Alvin* dives targeted previously explored sampling areas (Teske et al., 2016), or newly identified sites found by AUV *Sentry*. After *Sentry* returned from pre-programmed night dives at ca. 6AM, dive data and bathymetries were downloaded and made available in time for the following *Alvin* dive starting at 8AM. When *Sentry* performed seafloor photomosaic surveys running ca. 6m above bottom, the resulting images were inspected for microbial mats and potential dive targets, for example, in the “Northern Towers” area of the southern axial trough of Guaymas Basin. Photo coverage of *Alvin* dives is available at the *Alvin* frame-grabber site.^1^ The bathymetry of the hydrothermally active graben segment in southern Guaymas Basin was mapped by AUV *Sentry* during dives 407-409, 413-417; the bathymetry of Octopus Mound at the Central Seep site was mapped during *Sentry* dive 412 (Figure 1). Survey height was 65–70m above the bottom. *Alvin* sampling sites were largely based on *Sentry* surveys (Supplementary Figure S1).

### Thermal Profiles

Thermal profiles were measured in surficial sediments using *Alvin’s* 50cm or 1-m heat flow probes.^2^ The 50cm probe was used for hydrothermal sites in the southern axial valley and contains thermal sensors every 10cm, starting 5cm under the attached plastic disk (the “puck”) that limits probe penetration and rests on the seafloor once the probe was inserted. Cold seep sediments of Octopus Mound were profiled using the 1-m probe with thermal sensors every 20cm. After 5–10min of temperature reading stabilization, temperature readings were recorded. Thermal profiles adjacent to sediment cores that were analyzed in this study are shown in Supplementary Figure S3.

### DNA Extraction, Library Preparation, and High-Throughput 16S rRNA Gene Sequencing

DNA was collected from the top 0–1cm of sediment cores after removal of any overlying microbial mat, except for one mat-covered core (4862-08) where draining fluids had sucked the mat into the sediment before slicing. Genomic DNA was extracted from 0.5g of sediment from each sample using the DNeasy PowerLyzer PowerSoil Kit (Cat. No. 12855-100, QIAGEN) and bead-beating at 6ms^−1^ for 45s using a Bead Ruptor 24 (OMNI International, Kennesaw, GA, United States). Extraction blanks were performed alongside the samples to assess laboratory contamination during the extraction process. DNA concentrations were assessed fluorometrically using a Qubit 2.0 fluorometer (Thermo Fisher Scientific, Canada). Bacterial 16S rRNA gene variable region v3-v4 was amplified using the “universal” primer pair 341F (5'-CCTACGGGAGGCAGCAG-3'; Klindworth et al., 2013) and Pro805R (5'-GACTACNVGGGTATCTAATCC-3'; Takahashi et al., 2014). The archaeal 16S rRNA gene v4-v5 variable region was amplified using the combined forward primers 517F (5'-GCCTAAAGCATCCGTAGC-3', 5'-GCCTAAARCGTYCGTAGC-3', 5'-GTCTAAAGGGTCYGTAGC-3', 5'-GCTTAAAGNGTYCGTAGC-3', 5'-GTCTAAARCGYYCGTAGC-3') and reverse primer 958R (5'-CCGGCGTTGANTCCAATT-3'; Topçuoglu et al., 2016). The primers were modified with Illumina MiSeq overhang adapters. Each PCR reaction consisted of 1–5μl (~10ng) genomic DNA template, 2.5μl of each of the primers (final concentration 1μM), 12.5μl 2X Kapa HiFi HotStart ReadyMix (Kapa Biosystems, Wilmington, MA, United States) and sterile nuclease-free water to make a final volume of 25μl gene amplification was carried out using an initial degradation at 95°C for 3min following 25cycles of denaturation at 95°C for 30s, annealing at 56°C for 45s, and extension at 72°C for 1min, and concluded with a final extension at 72°C for 5min. All PCR reactions were performed in triplicate, pooled, and purified using the NucleoMag NGS Clean-up and Size Select kit (Macherey-Nagel Inc., Bethlehem, PA, United States). The purified PCR products were indexed following the instructions on Illumina’s 16S amplicon library preparation guide. The concentration of dsDNA and the size of the indexed amplicons were verified using the Qubit dsDNA High Sensitivity assay kit on a Qubit 2.0 fluorometer (Thermo Fisher Scientific, Canada) and the Agilent 2100 Bioanalyzer system (Agilent Technologies, Mississauga, ON, Canada), respectively. Indexed amplicons were pooled in equimolar amounts and sequenced using Illumina’s v3 600-cycle (paired-end) reagent kit on a MiSeq benchtop sequencer (Illumina Inc., San Diego, CA, United States).

### Microbial Community Analyses

Raw bacterial 16S rRNA gene amplicon sequence data were analyzed using MetaAmp (Dong et al., 2017), and archaeal raw sequence data were analyzed using DADA2 (Callaghan et al., 2016). Taxonomy of operational taxonomic units (bacterial OTUs, defined at 98% sequence identity) or amplicon sequence variants (archaeal ASVs) was assigned using the SILVA reference database v132 (Quast et al., 2013). Community analyses were performed using VisuaR^3^, an R-based workflow using the packages vegan, labdsv, tidyverse (stringr, dplyr, ggplot2), UpSetR, and custom scripts (Wickham, 2016, 2018; Oksanen et al., 2012; Roberts, 2012; Conway et al., 2017; Wickham et al., 2018). The original OTU or ASV abundance tables were used to calculate richness and diversity indices, i.e., Inverse Simpson diversity (Hill et al., 2003), Shannon entropy, and Chao1 estimated richness (Chao, 1984) with a subsampling approach to ensure comparability of indices. Dissimilarities between samples were calculated using the Bray-Curtis dissimilarity coefficient (i.e., relative sequence abundance; Bray and Curtis, 1957). The resulting beta-diversity matrices were used for 2D non-metric multidimensional scaling (NMDS) ordinations with 20 random starts (Kruskal, 1964). Stress values below 0.2 indicate that the multidimensional dataset is well represented by the 2D ordination.

### 16S rRNA Gene Phylogeny

Phylogenetic placement of amplicon sequences was conducted using the SILVA database SSU Ref NR 132 (Quast et al., 2013) and the software ARB (Ludwig et al., 2004). ASVs were first added to the SILVA tree using the ARB “quick add” tool, neighboring near full-length sequences (>1,300 nucleotides) were selected and aligned using SINA (Prüsse et al., 2012). The alignment was manually curated based on ribosomal secondary structure and was subsequently used to calculate 100 maximum likelihood phylogenetic trees with the phyML algorithm, of which the most likely tree was automatically selected. We used a positional variability filter including only conserved positions in the alignment with a mutation rate of <3.1%. Finally, amplicon sequences were added to the consensus tree using the same positional variability filter without changing the overall tree topology.

### DNA Isolation and *mcrA* Gene Sequencing

A second set of DNA extractions from sediment was performed with the MO BIO PowerSoil DNA Isolation Kit (QIAGEN, Carlsbad, CA, United States) for the purpose of *mcrA* gene amplification and sequencing. Amplification was performed with general *mcrA* primer combination mcrIRD-F (5'-GACCAGTTGTGGTTCGGAAC-3') and mcrIRD-R (5'-ATCTCGAATGGCATTCCCTC-3'; Lever and Teske, 2015). The PCR protocol contained 30cycles of initial denaturation at 95°C for 1min, annealing at 55°C for 1min and extension at 72°C for 1min, and concluded with a final extension at 72°C for 5min. PCR products were purified using the Wizard SV Gel and PCR Cleanup System (Promega Corporation, Madison, WI, United States) and cloned into plasmid vectors using the TOPO TA Cloning Kit (Life Technologies, Carlsbad, CA, United States). These were used to transform One Shot TOP10 *Escherichia coli* cells (Life Technologies, Carlsbad, CA, United States) which were plated on selective media. Approximately 25 colonies per sample were picked and incubated overnight in SOC medium, then plasmids were extracted using the GeneJET Plasmid Miniprep Kit (Thermo Fisher Scientific, Waltham, MA, United States). Plasmids were then sent to GeneWiz (South Plainfield, NJ, United States) for sequencing. The alignment of representative *mcrA* gene sequences was created in the MEGA software using the MUSCLE algorithm (Edgar, 2004), and the tree was created from the gene sequence alignment using the neighbor-joining method in MEGA4 (Tamura et al., 2007). The topology of the *mcrA* phylogeny was tested by 500 bootstrap runs.

### Porewater Geochemistry

For porewater analysis, intact sediment cores were sampled using the Rhizons (Rhizosphere Research Products, Wageningen, NL) as described previously (Seeberg-Elverfeldt et al., 2005). The overlying water was removed from the cores. Holes were drilled into the core at designated sediment sampling depths and pretreated Rhizons (washed twice with HCl and MilliQ water) were injected and suction was applied with syringes for ~30min. For sulfide analysis, 1ml porewater subsamples were fixed with 0.1ml of 0.1M zinc acetate solution to preserve sulfide as zinc sulfide until analysis using the methylene blue method (Cline, 1969). The same fixed porewater sample was used for measuring sulfate concentrations using ion chromatography (Metrohm 930 Compact IC flex oven, Metrosep A PCC HC/4.0 preconcentration column, and Metrosep A Supp 5 Guard/4.0 chromatography column). The concentrations of ammonium, phosphate, and silicate were determined from the same porewater samples using a continuous flow nutrient analyzer (QuAAtro39; Seal Analytical) as published previously (Grasshoff et al., 2009). For combined concentration and *δ*^13^C analysis of methane, 2ml sediment subsamples were added to 30ml serum vials containing 2ml of 1M sodium hydroxide solution, sealed with thick butyl rubber stoppers, crimped with aluminum seals, and stored at 4°C. Shipping problems and a resulting shortage of serum vials limited sampling capabilities and only selected sediment cores were sampled for methane. Since cores were retrieved unpressurized, outgassing may have impacted in particular the measurements of methane concentrations near and above saturation. After the cruise, the methane samples were analyzed by headspace gas chromatography-flame ionization detection (GC-FID) at Florida State University (Magen et al., 2014). Additionally, the gas samples were analyzed for *δ*^13^CH_4_ by injecting 0.1–0.5ml of sample into a gas chromatograph interfaced to a Finnigan MAT Delta S isotope ratio Mass Spectrometer inlet system as previously described (Chanton and Liptay, 2000). Values are reported in the per mil (‰) notation relative to Vienna Pee Dee Belemnite (VPDB).

## Results

### Guaymas Basin Survey Sites

During expedition AT37-06 to Guaymas Basin, AUV *Sentry* and submersible *Alvin* mapped and sampled hydrothermally active sediments within the southern Guaymas Basin spreading center, at an off-axis sill-driven vent site (Ringvent) with hybrid seep/vent biota on the northwestern flanking regions near Isla Tortuga (Teske et al., 2019), and at the off-axis Central Seep site on the northwestern Guaymas flanks (Geilert et al., 2018) that is approximately equidistant from the Sonora and Baja California coasts (Figure 1).

**Figure 2:**
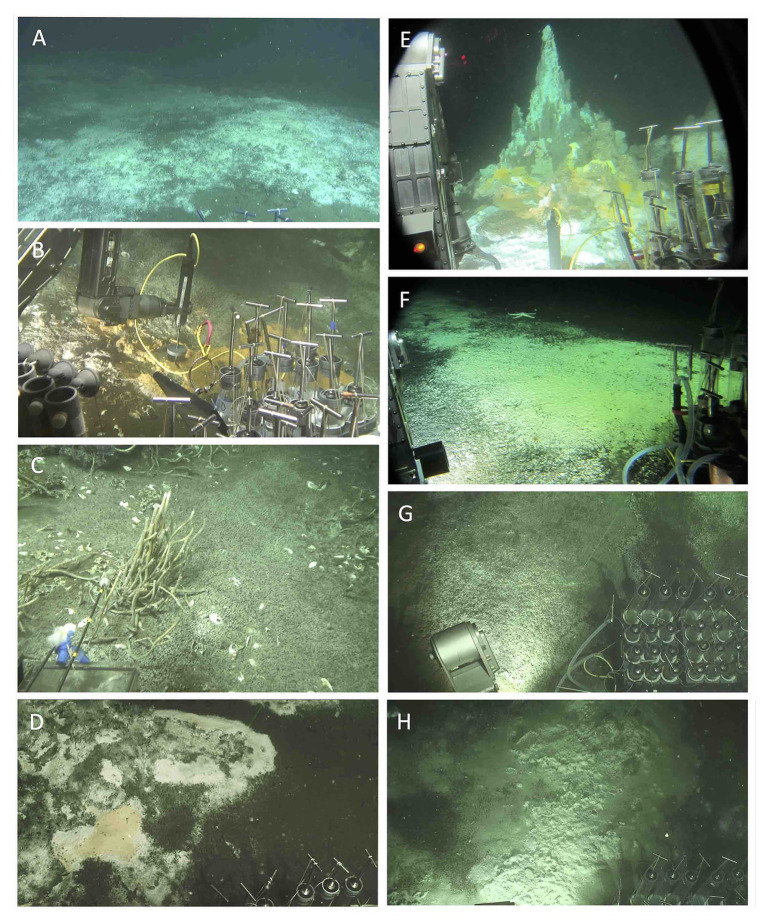
Sampling sites for microbial and/or biogeochemical analyses. **(A)** Extensive white mat at Mat Mound Massif. Dive 4861. **(B)** Hydrothermal sediment with orange *Beggiatoaceae* mat, Mat Mound Massif. Dive 4862. **(C)** Cold seep site “active site” at Octopus Mound. Dive 4867. **(D)** Hydrothermal sediment with orange *Beggiatoaceae* mat at Mat Mound Massif. Dive 4869. **(E)** Summit and slopes at Cathedral Hill. The cores are from the white, fluffy mat area at the bottom of the photo. Dive 4870. **(F)** Temperate Aceto Balsamico mat with lime-yellow sulfur precipitates. Dive 4870. **(G)** Temperate “site 2” mat-covered sediment at Northern Towers. Dive 4871. **(H)** Hot “site 3” mat-covered hydrothermal sediment at Northern Towers. Dive 4871. The sites were photographed from inside *Alvin* (dives 4862, 4867, 4870) or documented as *Alvin* screen grabber images (dives 4861, 4869, 4871). Images courtesy of *Alvin* group, WHOI.

Different types of mat-covered sediments and thermal regimes were sampled at these locations (Figure 2). The Mat Mound Massif, a cluster of hydrothermal mounds and edifices, was probed from different angles on previous cruises (e.g., Dowell et al., 2016) but its full extent was only recognized during this expedition. The Aceto Balsamico area is located ca. 150m west of Mat Mound Massif and contains moderately warm sediments covered with lime-yellow mats of sulfur-oxidizing bacteria (Teske et al., 2016). At Cathedral Hill, ca. 200m north of Aceto Balsamico, gradually sloping sediment-covered mounds with extensive microbial mats are topped with hydrothermal edifices (Teske et al., 2016). The Northern Towers area is located ca. 5km northeast from the other, tightly clustered sites, and was not sampled during previous expeditions in 2008/2009 (Teske et al., 2016). It has relatively few hydrothermal sediments and mats but is dominated by massive, steep hydrothermal edifices and chimneys. Here, microbial mats were located by AUV *Sentry* in photomosaic survey mode, and their coordinates were targeted during subsequent *Alvin* dives. *Alvin* push cores from all locations were collected during Expedition AT37-06 on *Alvin* dives 4861, 4862, 4867, 4869, 4870, and 4871 (Table 1).

**Figure 3:**
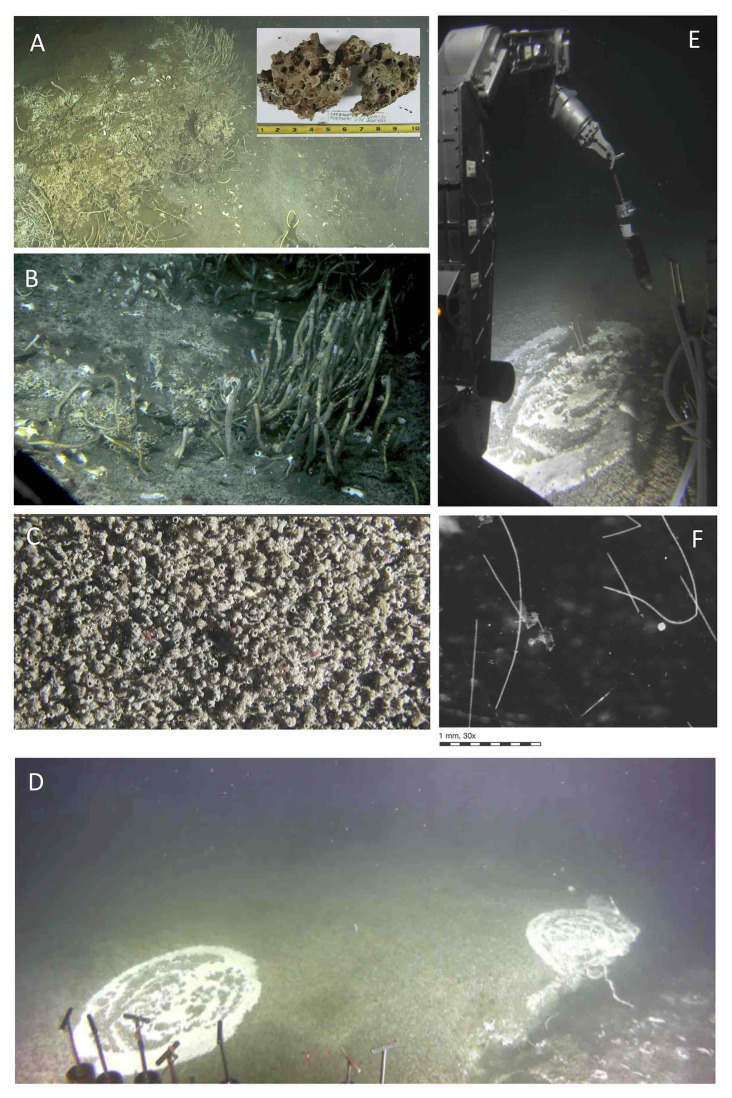
Benthic fauna at Octopus Mound sampling sites. **(A)** Seafloor carbonate concretions with tube worms at the “active site” near the base of Octopus Mound. Insert, carbonate sample collected at this location during *Alvin* dive 4867. **(B)** Seep community with different types of tubeworms resembling *Lamellibrachia*, galatheid crabs, and clam shells. The original image was underexposed and had to be digitally manipulated, resulting in over-emphasized steel-blue hues instead of olive-green and brown tones. **(C)** Close-up view of ampharetid worm carpet, with pink worms protruding from several worm tubes. Overall view ~10 × 20cm; video still from *Alvin’s* bottom camera. **(D)** Sedimented Hydrate mound, overgrown with an extensive mat of ampharetid worms and circular spots of *Beggiatoaceae* mats. At the massive fracture to the right, the mat-covered sediment is ca. 0.5m elevated, suggesting rising hydrate underneath. The *Beggiatoaceae* mat to the left was sampled with *Alvin* push cores. **(E)** Coring the *Beggiatoaceae* mat on top of the hydrate mound during dive 4867, the “hydrate site.” The bottom of the freshly collected core contains white gas hydrate (presumably methane hydrate) that dissipates during transport to the surface. **(F)** Individual *Beggiatoaceae* filaments recovered from the hydrate mat are viewed through a dissection binocular. Filament diameters are in the range of 100–160μm, consistent with large, colorless *Beggiatoaceae* observed previously in Guaymas Basin (McKay et al., 2012; Teske and Salman, 2014). Images A–E courtesy of the *Alvin* group, WHOI; dissection scope image of filaments by Barbara MacGregor.

Bathymetric mapping with AUV *Sentry* and reconnaissance with submersible *Alvin* at the Central Seep location revealed a mound of 200m north-to-south and 100m east-to-west extension that rises ca. 20m above the seafloor. The base of the mound at its northern end, and nearby sediments harbored extensive cold seep communities and surface-breaching gas hydrates (Figure 3). This mound was informally named “Octopus Mound” for its abundance of cephalopods (Supplementary Figure S1) after it was explored and sampled during *Alvin* dives 4866 and 4867 (December 17–18, 2016). Surface sediment samples were collected by HOV *Alvin* within ~100m of each other at the northern tip of Octopus Mound in an area with small-scale topographical diversity, diverse cold seep fauna, seafloor mineral formations, microbial mats, and hydrates (Figure 3). Sampling locations at Octopus Mound (Supplementary Table S1) were chosen based on the presence or absence of seep fauna assemblages (Supplementary Figure S2).

### Porewater and Thermal Profiles

**Figure 4:**
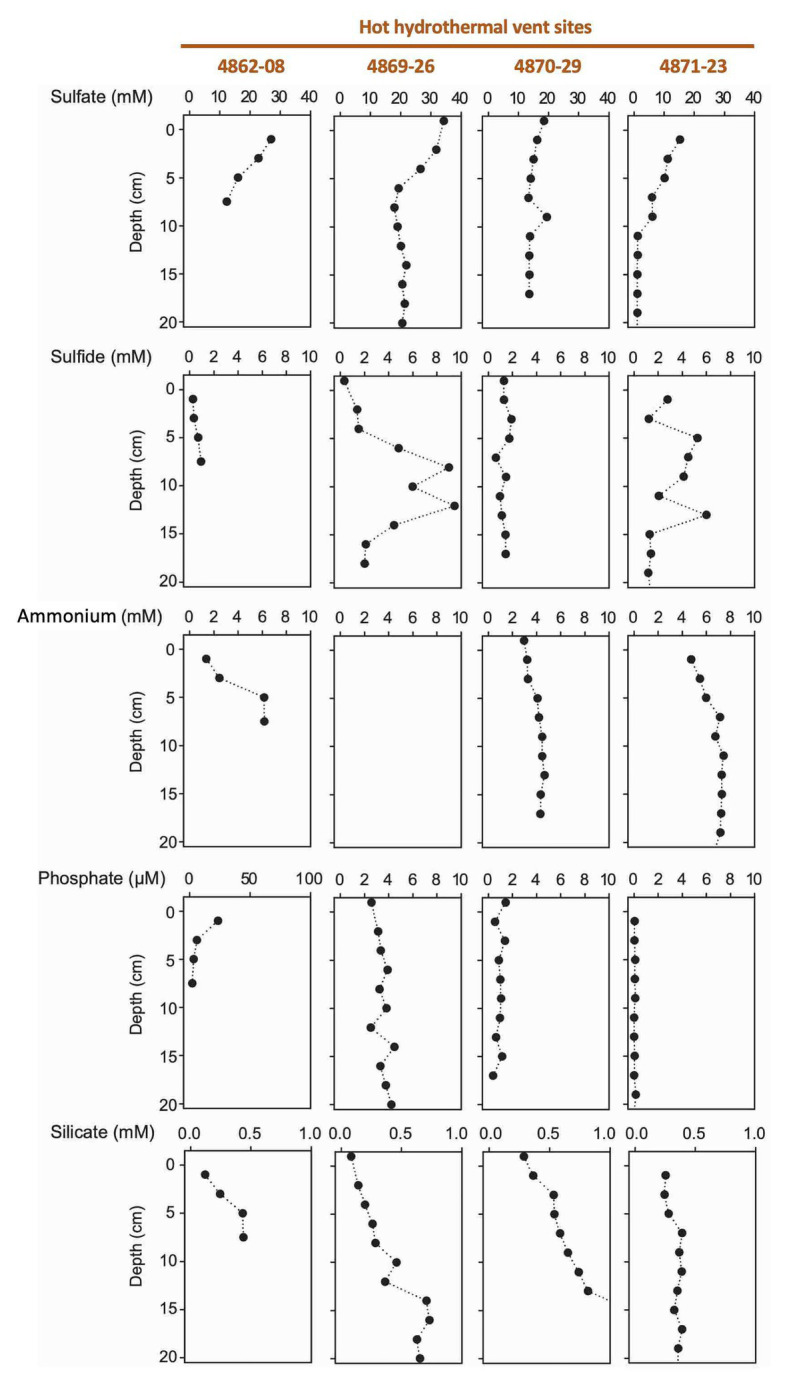
Porewater concentration profiles of sulfate, sulfide, ammonium, phosphate, and silicate in four hydrothermal cores.

**Figure 5:**
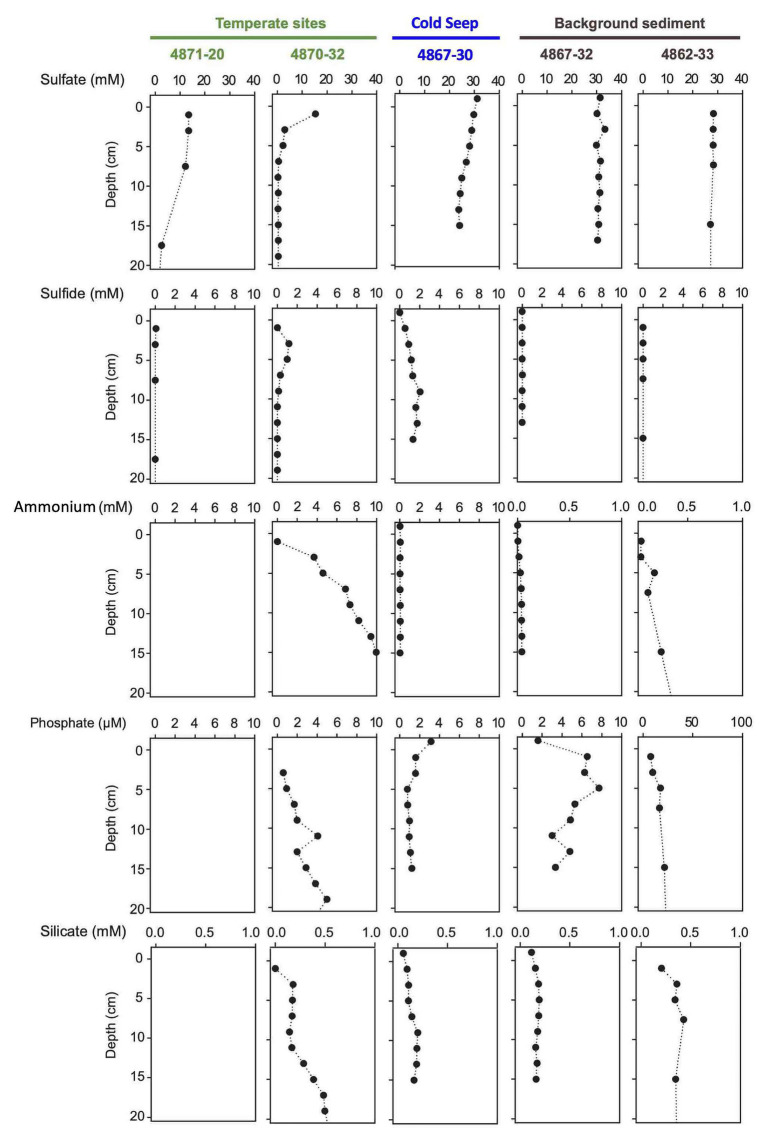
Porewater concentration profiles of sulfate, sulfide, ammonium, phosphate, and silicate in two temperate cores, one seep core, and two background cores without visible microbial mats. Empty panels indicate data gaps.

The Guaymas Basin cores from these diverse sampling locations can be grouped into three categories based on thermal profiles and microbial mat cover: hot hydrothermal sediments with conspicuous microbial mats and temperatures reaching >50°C within 50cm depth, temperate hydrothermal sediments with microbial mats and a temperature range between 5 and 50°C, and background sediments without visible mats, and a temperature range of 3–5°C (Supplementary Tables S2 and S3, Supplementary Figures S2, S3). These categories were also reflected in the porewater profiles (Figures 4, 5).

#### Hydrothermal Sediments

The hot hydrothermal sediments (cores 4862-08, 4869-26, 4870-29, 4871-23) are characterized by steeply increasing temperatures into the range of >80°C by 50cm depth (Supplementary Table S1, Supplementary Figure S3) and thick microbial mats covering the seafloor (Supplementary Figure S2). The extracted porewater contained high sulfide (1–10mM) and ammonium (up to 7mM) concentrations (Figure 4). Elevated concentrations of dissolved silicate (>0.2mM) suggest hydrothermal dissolution and mobilization of solid silicate phases, and irregular sulfate profiles most likely represent seawater inmixing (Figure 4). Cores in the “temperate” sediment category (cores 4870-32 and 4871-20) had moderate *in situ* temperatures of 10–30°C. The ammonium concentration of core 4870-32 (5–10mM; no ammonium data for core 4871-20) was even higher than observed at the hot hydrothermal sites. Sulfate is rapidly depleted below the sediment surface, indicating the absence of seawater sulfate inmixing; sulfide appears to be limited to surficial sediments (Figure 5). In the “background” category, *Alvin* cores 4862-33 and 4867-32 have temperatures near bottom seawater (3–4°C). These cores have significantly lower ammonium and silicate concentrations, in combination with near-seawater sulfate concentrations; sulfide was only found in micromolar traces (Figure 4B). Sediment core 4867-30 from the off-axis Octopus Mound sampling site does not fit easily into these categories; it is uniformly at bottom water temperature (2.9°C), lacks hydrothermal signatures such as high ammonium or silicate concentrations, and shows only moderate sulfate depletion; yet the core was strongly sulfidic (Figure 5).

#### Cold Seep Sites

*Alvin* dives 4866 and 4867 provided an opportunity to inspect different faunal assemblages and geochemical settings, and to collect sediment push cores from closely spaced targets at the edge of Octopus Mound. As determined with the *Alvin* heat flow probe, all sampling sites at Octopus Mound had *in situ* temperatures between 2.9°C at the seawater interface, increasing towards local maxima of 2.95–3.03°C at different shallow sediment depths as determined with the *Alvin* heat flow probe (Supplementary Table S3). The “active site,” named for its conspicuous benthic invertebrate community, harbors a cold seep assemblage of tube worms, seep clams, and dense populations of ampharetid worms covering the sediment surface (Figures 3A,B,C). Sampling holes after core removal were deep black, indicative of sulfidic, strongly reducing conditions. The geochemically analyzed core 4867-30 was collected at this location. The “periphery” site was selected to sample the edge of this conspicuous seep community, at a distance of ~2m. The “background” site lacked visible seep fauna. The “gas hydrate site” (Figures 3D,E,F) was initially sampled for its conspicuous circular microbial mat consisting of large, filamentous sulfur-oxidizing bacteria of the family *Beggiatoaceae* (Teske and Salman, 2014), with ca. 100 μm filament diameter (Figure 3F). On closer inspection, the sulfur-oxidizing *Beggiatoaceae* mat was growing on top of an ampharetid worm assemblage that was spreading over the entire hydrate area (Figures 3D,E). When push cores were removed from the sediment, the bottom end contained white hydrates, presumably methane hydrate, which dissociated during transport to the surface (Figure 3E). Sequence-based and geochemical analyses for these cores are briefly summarized in Supplementary Table S1.

Porewater nutrient profiles set the Octopus Mound sediments apart from their hydrothermal counterparts (Figure 5). The concentrations of ammonium were near 50 μM (“active” core 4867-30 and “background” core 4867-32), approximately two orders of magnitudes lower than measured in porewaters from the hydrothermal sites. Independently obtained ammonium profiles from nearby multicorer and gravity cores collected by the R/V *Sonne* expedition in 2015 yielded similar ammonium concentrations, around 30 μM in surficial sediments, and increasing to 300 μM at 4m depth (Geilert et al., 2018). Phosphate concentrations remained below 2 μm in the active core and 10 μm in the background core sediments, respectively. Porewater silicate concentrations in the *Alvin* push cores were in the range of 100–200 μm, near the bottom water concentration of ca. 175 μM in Guaymas Basin (Campbell and Gieskes, 1984), and much below the elevated dissolved silicate concentrations of 0.5–2mM that characterize the porewater of hydrothermal Guaymas Basin cores (Figure 5).

**Figure 6:**
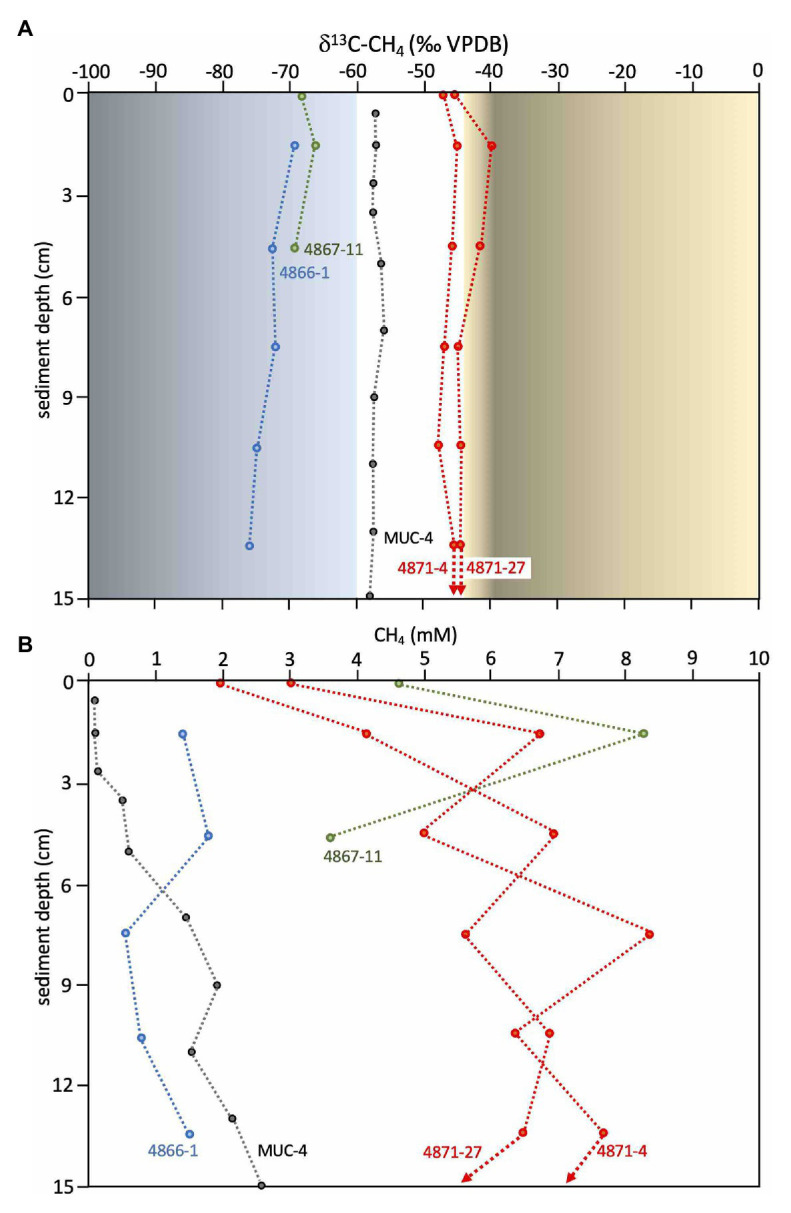
Methane isotopic values and concentrations. **(A)** Methane *δ*^13^CH_4_ values (VPDB) for Octopus Mound cores 4866-1 (active site, in blue) and 4867-11 (hydrate site, in green), and the nearby sediment push core MUC-4 (black), previously collected and measured independently at Central Seep (Geilert et al., 2018). Methane *δ*^13^CH_4_ values (VPDB) for hydrothermal cores from the Northern Towers area (4871-4 and 4871-27) are plotted in red. Orange shading indicates the range of *δ*^13^CH_4_ values for thermogenic methane in hydrothermal sediments in the southern Guaymas axial valley (McKay et al., 2016). Blue shading indicates the range of *δ*^13^CH_4_ values for microbially produced methane in cold sediments of the Sonora Margin (Vigneron et al., 2015, Teske et al., 2019), delimited at 60‰ based on Schoell (1982) and Simoneit et al. (1986). **(B)** Methane concentrations for the same samples. Data points for supernatant samples are plotted at 0cm depth. *δ*^13^CH_4_ values and their standard deviations, and methane concentrations are tabulated in Supplementary Table S4.

Methane at Octopus Mound is predominantly biogenic, as indicated by Octopus Mound *δ*^13^C-CH_4_ values from −68 to −76‰ (Figure 6A), falling between the biogenic methane of Sonora Margin cold seeps (ca. −80 ‰, Vigneron et al., 2015) and the mixed biogenic/thermogenic methane at the off-axis Ringvent site (ca. −60 ‰, Teske et al., 2019). Methane concentrations in the Octopus Mound sediment cores were often supersaturated, ranging from 0.6 to 1.8mM at the active site, and between 3.6 and 8.3mM at the hydrate site (Figure 6B). Methane concentrations in sediments at the periphery and background sampling areas were below detection. A comparison to hydrothermal conditions is provided by two hydrothermal cores from the Northern Tower area, 4871-04 and 4871-27 (Figure 6A). Due to data gaps, these two cores were not included in broader biogeochemical and microbiological comparisons, but their microbial mat cover and their temperature maxima >100°C (Supplementary Table S2) identify them as hydrothermal. Their *δ*^13^C-CH_4_ profiles approach the values of previously studied hydrothermal cores dominated by thermogenic methane, and their multi-millimolar methane concentrations are typical for hydrothermal methane-saturated sediments of Guaymas Basin (McKay et al., 2016). As illustrated by this comparison, biogenic methane dominates at Octopus Mound, whereas thermogenic methane is either entirely absent or limited to a minimal contribution.

### Microbial Community Structure

**Figure 7:**
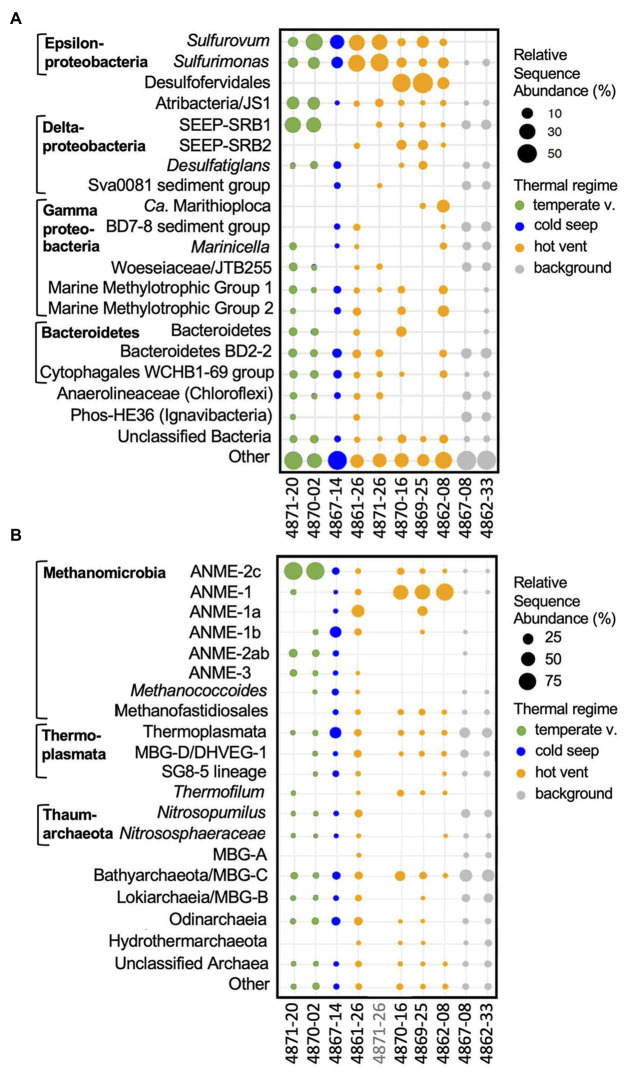
Bacterial and archaeal community composition of Guaymas Basin sediment cores. The size of the dots indicates the relative sequence abundance of microbial clades based on 16S rRNA gene amplicon sequences. The 20 most abundant bacterial **(A)** and archaeal **(B)** family-level lineages are shown. Less abundant clades are summarized as “Other.” When appropriate, taxa are annotated to supplement the automated SILVA identifications. DNA from core 4871-26 had run out after several sequencing attempts before archaeal sequencing could be finalized.

The taxonomic composition of bacterial and archaeal 16S rRNA gene sequences recovered from surficial sediments is distinctly different for hot hydrothermal sediments, temperate hydrothermal sediments, and background sediments (Figure 7). All sequence-based analyses have to be qualified by the fact that they are based on sequence frequencies, which are derived from the microbial community but do not necessarily represent it in identical proportions, and do not provide independent quantifications.

#### Hot Hydrothermal Sediment

Sequences of the sulfur-oxidizing chemoautotrophic genera *Sulfurovum* and *Sulfurimonas* within the Epsilonproteobacteria (Campbell et al., 2006) are broadly shared among sulfide-rich hydrothermal sediment cores of Guaymas Basin (Figure 7A). Mat-forming filamentous sulfur-oxidizing bacteria of the family *Beggiatoaceae* were abundant *in situ* (Figure 2), but the mats were removed for separate analyses before sediment slicing and DNA extraction. Therefore, *Beggiatoaceae* sequences (here assigned to *Ca*. Marithioploca, Figure 7A) were found in high abundance only in a single core (core 4862-08) where draining fluids had sucked the overlying mat into the surficial sediment. Sulfate-reducing bacteria are dominated by thermophiles: Sequences of the *Ca*. Desulfofervidus auxilii lineage occur in high frequency in the hot hydrothermal cores 4869-25 and 4870-16, and in reduced proportions in hydrothermal sediment core 4862-08. *Ca*. Desulfofervidus is the thermophilic bacterial syntroph of methane- and short-chain alkane-oxidizing archaea that dominates in enrichment cultures at temperatures of 37–60°C (Holler et al., 2011; Laso-Pérez et al., 2016; Krukenberg et al., 2018; Hahn et al., 2020). Interestingly, *Ca*. Desulfofervidus sequences were not recovered from core 4861-26, collected from an extensive mat area covered with white sulfur precipitates (Figure 2A) The intermediate temperatures there (T max. near 50°C, Table 1) may not suffice to sustain *Ca*. Desulfofervidus populations; the core smelled strongly sulfidic upon shipboard recovery but detailed geochemical data are not available. Sequences assigned to mesophilic sulfate-reducing bacteria of the SEEP-SRB1 cluster within the *Desulfobacteraceae* (Schreiber et al., 2010) account for smaller proportions of hydrothermal core sequences, and are absent in 4861-25. Sulfate-reducing bacteria of the SEEP-SRB2 cluster, an uncultured, presumably mesophilic and syntrophic lineage that may participate in short-chain alkane oxidation (Kleindienst et al., 2012; Krukenberg et al., 2018), occur in four out of five of the hot hydrothermal cores, except 4871-26 (Figure 7A). Heterotrophic, anaerobic phyla with fermentative potential (Atribacteria, Bacteroidetes, Chloroflexi) are represented by sequences from the hot hydrothermal cores, but were generally recovered from the temperature cores and background sediments (Figure 7A). Sequences of aerobic methylotroph MMG1 and MMG2 groups (Ruff et al., 2013) occur in hot and temperate hydrothermal cores (Figure 7A).

Sequences assigned to the dominant archaeal group, ANME-1, were recovered at the highest relative abundance from the fully mat-covered cores 4862-08, 4869-25, and 4870-16 (Figure 7B); other anaerobic methane-oxidizing archaea (ANME) types were found as well (most consistently, ANME-2c) but appear more frequently in the temperate hydrothermal cores. The most consistently found methanogenic group are methylotrophic *Methanofastidiosales* (Nobu et al., 2016b), previously detected as “Guaymas euryarchaeotal group” in Guaymas Basin hydrothermal sediments (Dhillon et al., 2005). Crenarchaeotal sequences assigned to the heterotrophic, thermophilic sulfur-reducing, and moderately acidophilic genus *Thermofilum* (Zillig et al., 1983) occur in smaller but consistently detected proportions. Uncultured sediment-associated archaea within the Thermoplasmatales, and the Bathyarchaeota and Asgardarchaeota are found in the hot and the temperate hydrothermal cores (Figure 7B).

#### Temperate Hydrothermal Sediment

These sediments share the epsilonproteobacterial sequences of the high-temperature hydrothermal cores, but they consistently lack *Ca.* Desulfofervidus (Figure 7A). Surface samples of cool cores 4870-2 and 4871-20 are characterized by frequent recovery of sequences attributed to the sulfate-reducing bacterial SEEP-SRB1 group (Knittel et al., 2003) which include the mesophilic syntrophic partners of ANME-1 methane-oxidizing archaea (Schreiber et al., 2010), and to the fermentative, mesophilic Atribacteria (Nobu et al., 2016a; Katayama et al., 2020) which are common inhabitants of cold seeps (Ruff et al., 2015; Chakraborty et al., 2020) and anaerobic subsurface sediments (Starnawski et al., 2017). Sequences from the aromatics-degrading sulfate-reducing *Desulfatiglans* lineage were recovered from all temperate cores, as well as from several hot hydrothermal and background samples, consistent with its cosmopolitan distribution (Jochum et al., 2018). The archaeal sequences recovered were predominantly assigned to sulfate-dependent ANME-2c methane oxidizers for the Northern Towers and Aceto Balsamico cores, and ANME-1b methane oxidizers for the Octopus Mound core (Figure 7B). Most other archaeal groups are shared with the background sediment, including members of the Bathyarchaeia, Thermoplasmata, Lokiarchaea, Marine Benthic Group D (renamed Thermoprofundales, Zhou et al., 2019), the uncultured SG8-5 Thermoplasmatales lineage found in estuarine sediments (Lazar et al., 2017), Thaumarchaeota of the aerobic, ammonia-oxidizing families *Nitrosopumilaceae* and *Nitrososphaeraceae* (Stieglmeier et al., 2014), methylotrophic methanogens of the uncultured *Methanofastidiosales* (Nobu et al., 2016b) and of the genus *Methanococcoides* (Liu and Whitman, 2008), and small proportions of ANME-2ab archaea. Some of these widely shared archaeal sequences show a comparatively spotty occurrence pattern in the hot hydrothermal samples, suggesting that strong hydrothermal conditions are selecting against them (Figure 7B). A preference for moderate over hot hydrothermal conditions characterizes the Lokiarchaeota and Odinarchaeia within the Asgardarchaeota (Zaremba-Niedzwiedzka et al., 2017) that were found recently in metagenomic surveys of Guaymas Basin hydrothermal sediments (Dombrowski et al., 2018; Seitz et al., 2019).

#### Background Sediment

Sequences of the sulfur-oxidizing microaerobic and nitrate-reducing chemoautotrophic genus *Sulfurimonas* occurred in reduced relative abundance in the background cores 4862-33 and 4867-08; the related sulfur-reducing genus *Sulfurovum* was no longer detected (Figure 7A). Sulfate-reducing bacteria were represented by SEEP-SRB-1, *Desulfatiglans*, and the Sva0081 group of uncultured Desulfobacteraceae that are hypothesized to scavenge H_2_ in marine sediments (Dyksma et al., 2018). Uncultured marine sediment lineages that were frequently found include the deep-sea sediment lineage BD2-2 within the Bacteroidetes (Li et al., 1999) and the deep-sea sediment lineage BD7-8 within the Gammaproteobacteria (Li et al., 1999), the PHOS-HE36 clade (Dabert et al., 2001) within the Ignavibacteria (Iino et al., 2010), the cosmopolitan sediment-associated gammaproteobacterial JTB255 lineage (Woeseiaceae, Mussmann et al., 2017; Hoffmann et al., 2020), the WCHB1-69 lineage within the Cytophagales (Dojka et al., 1998), and diverse unclassified Anaerolineae. Based on the spotty detection of their sequences in vent and seep cores, these bacteria may tolerate some hydrothermal or seep activity, unless selected against by other extreme conditions, or they may be generally abundant in the surrounding sediment and thus occur as relic DNA in the seep cores. Sequences of aerobic methylotroph groups MMG1 and MMG2 (Ruff et al., 2013) were not detected (4867-08) or occurred in reduced proportions (4862-33; Figure 7A). The archaeal sequences resembled those from the temperate cores, with increased relative representation of the Bathyarchaeota and Thermoplasmatales, and the addition of Marine Benthic Group A, a sediment-dwelling sister lineage of the marine Thaumarchaeota (Lauer et al., 2016). Thus, the Guaymas Basin background sediments harbor the five globally distributed marine benthic archaeal groups of MBG-A, MBG-B (Lokiarchaeota), MBG-C (Bathyarchaeota), MBG-D (Thermoprofundales), and MBG-E (Thermoplasmata subgroup) that were originally discovered in cold North Atlantic seafloor sediments (Vetriani et al., 1999).

As previously observed for the geochemical characteristics of Octopus Mound sediments, the Octopus Mound seep core 4867-14 does not fit easily into these three categories; it differs from the background sediments by not having SEEP-SRB1 sequences among the 20 most frequently detected bacterial taxa (Figure 7A), from temperate sediments by its lower proportion of ANME-2c sequences, and from all sediments by its high frequency of ANME-1b sequences (Figure 7B).

### Community Clustering

**Figure 8:**
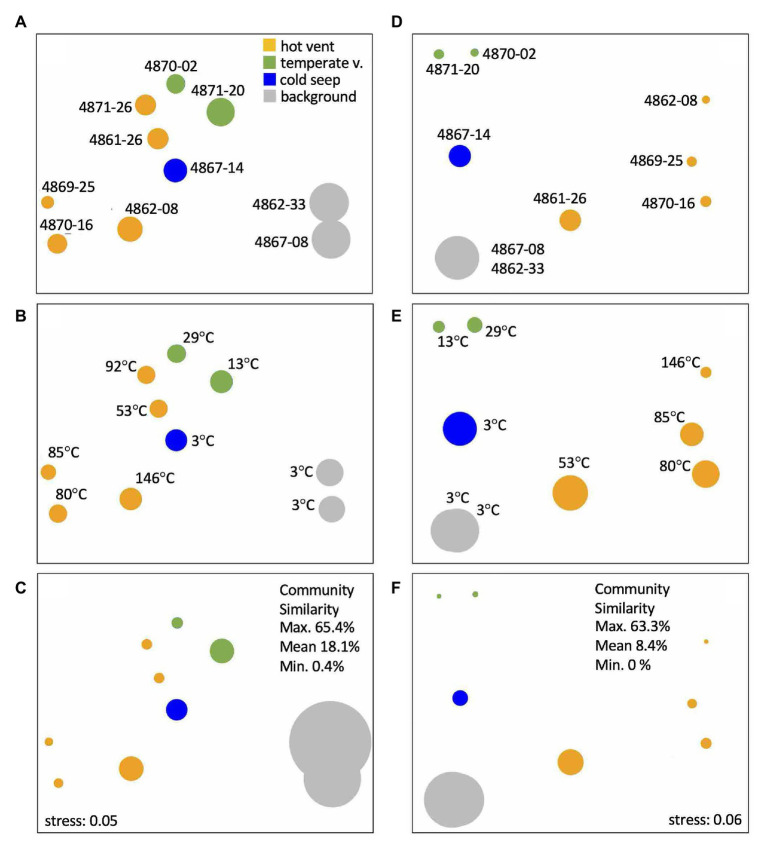
Non-metric multidimensional scaling (NMDS) ordination plots based on 16S rRNA gene amplicons for bacterial operational taxonomic units (OTUs; **A-C**) and archaeal amplicon sequence variants (ASVs; **D-F**) from the upper cm of sediment cores, color-coded by geochemical cluster, and annotated with core number **(A,D)** and *in situ* temperature at 50cm depth **(B,E)**. In plots **(A,D)**, circle size represents observed OTU richness. In plots **(B,E)**, circle size represents Shannon entropy. In plots **(C,F)**, circle size represents Inverse Simpson evenness.

Non-metric multidimensional scaling ordination plots show distinct clustering patterns for bacterial and archaeal communities that are consistent with geochemical characteristics (Figure 8). The strongest separation occurs between cold background cores 4862-33 and 4867-08, which cluster together in archaeal and bacterial NMDS despite their geographical distance of ca. 50km, and all other cores. Communities at hot and temperate sites are clearly separated from each other. The hot hydrothermal cores 4862-08, 4870-16, and 4869-25 stand apart from the temperate cores 4871-20, 4870-02, and the Octopus Mound core 4867-14, while the remaining hydrothermal cores (4871-26 and 4861-26) fall in between (Figure 8). Interestingly, core 4861-26 is the coolest of the hot hydrothermal cores, with a bottom temperature of 55°C (Supplementary Figure S3), and this intermediate thermal regime is consistent with its intermediate position in both archaeal and bacterial NMDS plots. Within the archaeal NMDS plots, the Octopus Mound core 4867-14 clusters apart from the cool hydrothermal sites (Figures 8D,E,F), consistent with its distinctive habitat characteristics. Except for the two background cores 4862-33 and 4867-08, no cores show overlapping NMDS clustering. These results suggest that sites with similar geochemistry can harbor microbial communities that diverge to some extent. Additional variables that were not measured could have an impact, or dynamic selection pressures due to rapidly changing hydrothermal regimes allow stochasticity to play an important role in site-specific community assembly (McKay et al., 2016). While these results contrast with a previous survey of Guaymas Basin microbial diversity that emphasized community overlap among hydrothermal sites within a constrained sampling area (Meyer et al., 2013), this survey covers a greater habitat range with distinct thermal and geochemical regimes, and thus distinct microbial communities.

### Microbial Methane Oxidation

**Figure 9:**
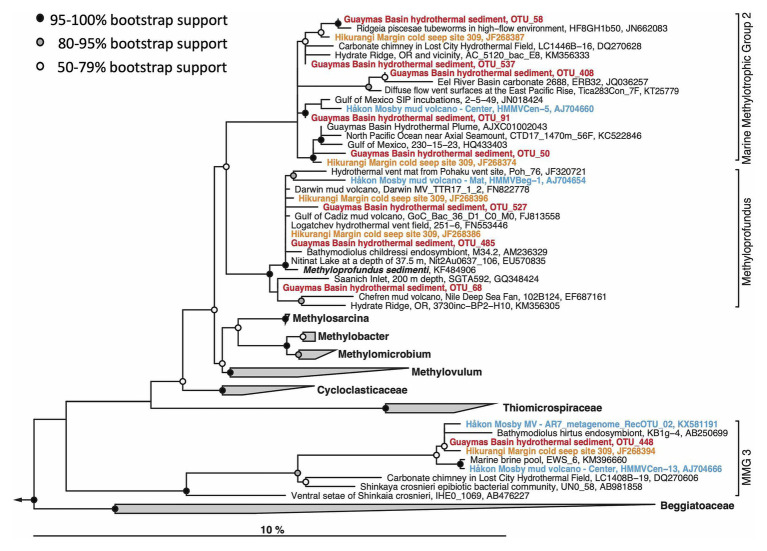
Maximum likelihood phylogeny of gammaproteobacterial methylotrophic and methanotrophic bacterial OTUs. The tree was calculated using near full-length sequences; partial 16S rRNA gene sequences (ca. 450 nucleotide positions) obtained with primers 341F and Pro805 were added without changing the tree topology. The *Methyloprofundus* branch is synonymous with Marine Methylotroph Group 1 (MMG1), a sister lineage to MMG2 and MMG3. Sequences used for the phylogeny are listed in Supplementary Table S5 for easy retrieval.

In contrast to previously sampled hydrothermal Guaymas Basin sediment communities, which were apparently dominated by ANME (Teske et al., 2002), the surface layer of the Octopus Mound site and several of the hydrothermal sites reported here have yielded 16S rRNA gene sequences of aerobic methanotrophic and methylotrophic bacteria (Figure 7A). Their detection could be linked to the sampling scheme; these aerobes are more likely to be detected in the top 0–1cm layer sampled here than in surficial samples of multiple centimeters that are predominantly anoxic. The sequences are affiliated with the cultured methanotrophic genus *Methyloprofundus* (Tavormina et al., 2015), and the uncultured lineages Marine Methylotrophic Groups 2 and 3 within the Gammaproteobacteria (Ruff et al., 2013). These groups contain gene sequences from New Zealand cold seeps, the Håkon-Mosby mud volcano, methylotrophic mussel endobionts, and diverse seafloor sediments (Figure 9). Some Guaymas Basin OTUs are specifically related to OTUs from methane-rich seeps and mud volcanoes, the Hikurangi Margin in New Zealand that harbors abundant amphetarid worm communities (Ruff et al., 2013), and the Håkon-Mosby Mud volcano in the Norwegian Arctic Ocean (Ruff et al., 2019).

**Figure 10:**
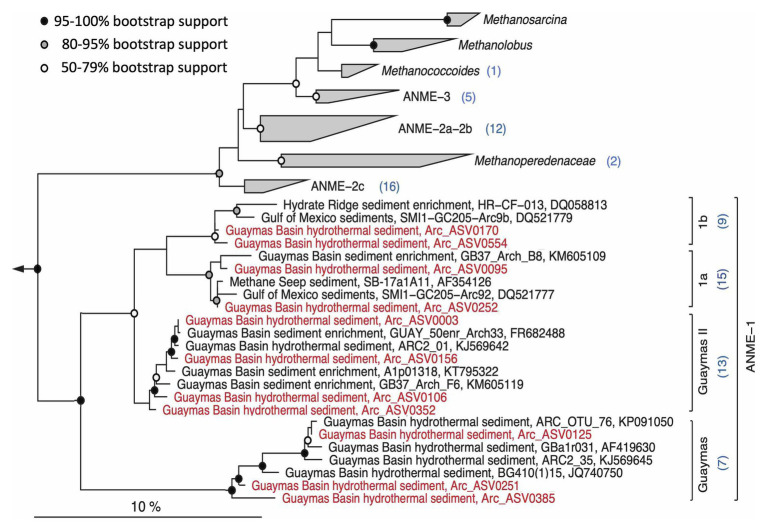
Maximum likelihood phylogeny of ANME-1 archaea and related ANMEs and methanogens. The tree was calculated using near full-length sequences; partial 16S rRNA gene sequences (ca. 450 nucleotide positions) obtained with primers 517F and 958R were added without changing the tree topology. The annotation numbers in parentheses indicate the number of representative ASVs for different clades. Sequences used for the phylogeny are listed in Supplementary Table S5 for easy retrieval.

Analogous to bacterial methanotrophs, the archaeal anaerobic methane oxidizers also show considerable phylogenetic complexity (Figure 10). Within the broadly defined ANME-1 archaea, some Guaymas ASVs were affiliated with ANME-1a and 1b, closely related subgroups that co-occur in diverse cold seeps (Knittel et al., 2005). The ASVs assigned by the SILVA pipeline to the generic “ANME-1” category (Figure 7B) are phylogenetically divergent and affiliate with two different ANME-1 lineages, the uncultured and presumably thermolerant ANME-1 Guaymas lineage that occurs consistently in hydrothermal Guaymas sediments (see data compilation in Dowell et al., 2016), and a cluster termed “ANME-1a Guaymas II” that was cultured in methane-oxidizing enrichments at 50°C (Holler et al., 2011) and also appears consistently in Guaymas hydrothermal sediments (Dowell et al., 2016). Thus, the ANME-1 archaea that are specifically recovered from hydrothermal cores (4862-08, 4869-25, and 4870-16) belong to lineages that are either cultured thermophiles or show a preference for hydrothermal sediments (Figure 10). In contrast, sequences affiliated with the diverse ANME-2 subgroups were recovered predominantly from temperate cores (Figure 6B), consistent with previous observations (McKay et al., 2016). The ANME-3 subgroup, previously identified in cold seep sediments of an arctic mud volcano (Niemann et al., 2006; Lösekann et al., 2007), is also preferentially found in temperate and cold seep sediments (Figure 7B).

**Figure 11:**
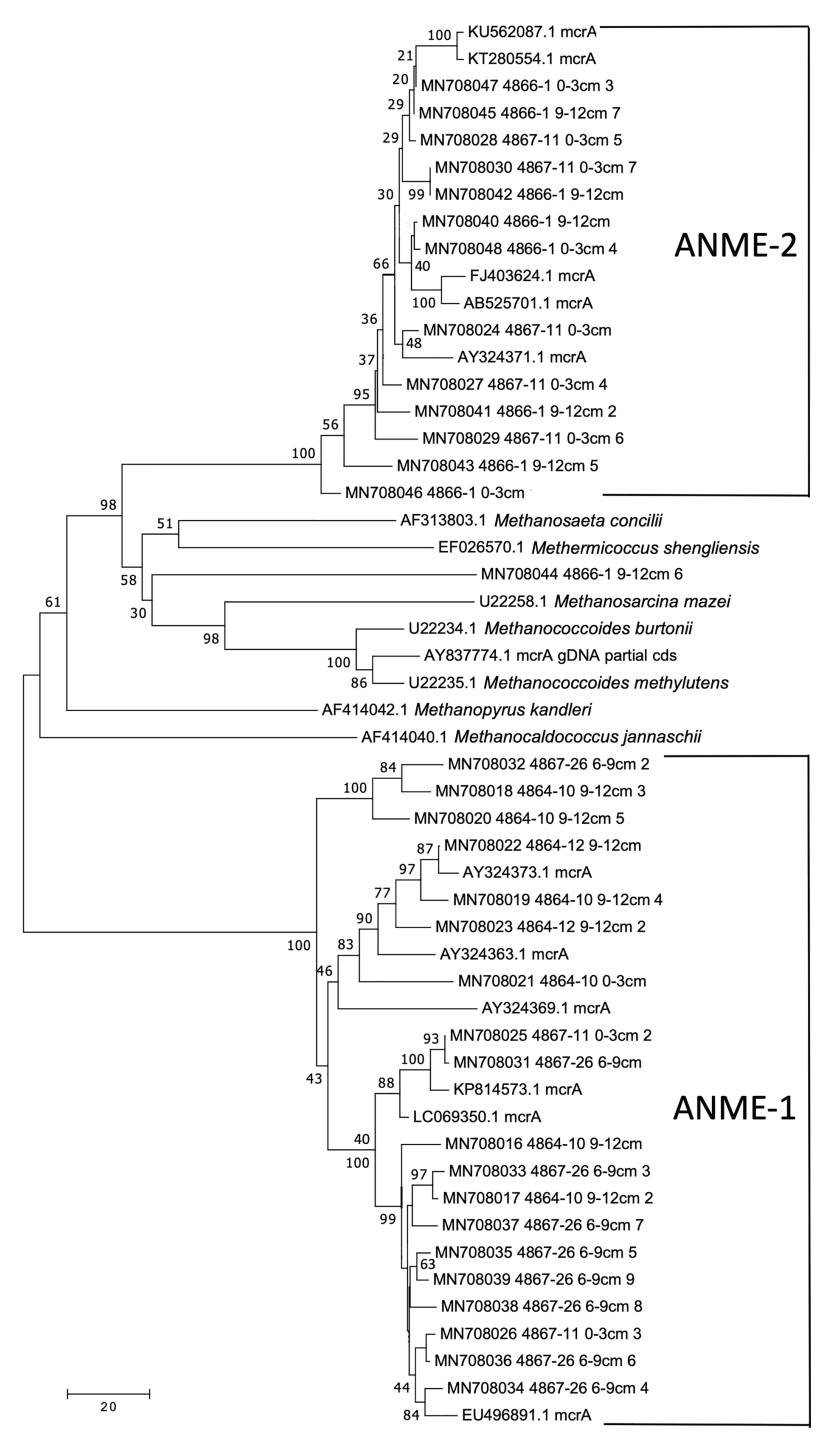
Distance phylogeny of *mcrA* genes recovered from Octopus Mound sediments. Taxon labels begin with the Genbank number, followed by the *Alvin* Dive and core number, and (if applicable) the number of multiple clones from the same location and core that is represented by this sequence. Additional *mcrA* genes come from Ringvent sediments sampled during *Alvin* dive 4864 (Teske et al., 2019). Bootstrap numbers were obtained by 500 replicates.

To further target anaerobic methanogenic and methane-oxidizing archaea at the Octopus Mound site, *mcrA* gene sequences that are diagnostic for these archaea were PCR-amplified and surveyed (Figure 11). The sediments at the ampharetid-dominated “active site,” sampled by *Alvin* core 4866-1, yielded *mcrA* sequences of the ANME-2ab clades; below-surface layers of core 4867-26 from the nearby “periphery site” yielded ANME-1; and the surficial centimeters of core 4867-11 from the hydrate site yielded ANME-1 and ANME-2 sequences (Figure 11). No *mcrA* gene sequences of cultured methanogenic genera or families were detected.

## Discussion

### Hydrothermalism vs. Cold Seepage

The strong contrasts between different Guaymas Basin hydrothermal sites and the Octopus Mound cold seeps in seafloor microbial community, benthic life, thermal characteristics, and porewater geochemistry highlight the diversity of geo-ecosystems in the greater Guaymas Basin region. A closer look at the thermal and biogeochemical parameters of these different sites shows that they are not always tightly coupled, suggesting underlying transitions or hybrids between these environmental regimes.

With ammonium concentrations in the 5–10mM range, silicate concentrations in the 0.5–2mM range, and high sulfide concentrations of 1–3mM, the hot hydrothermal sediment cores share key signatures of Guaymas Basin hydrothermal fluids (von Damm et al., 1985). Interestingly, these porewater indicators are not always linked to high temperatures. High ammonium and silicate concentrations are also observed in temperate hydrothermal sediments (core 4870-32 and 4871-20), suggesting that rising fluids with these hydrothermal signatures have cooled down locally before reaching the sediment surface. The lack of seawater sulfate below the upper 5cm of sediment (Figure 5) indicates that the sediments are not cooled by seawater inmixing near the surface. Instead, the hydrothermal fluids must have cooled down considerably during migration through the sediments. These conditions are not indicative of a transient regime, but remain stable over many years, as observed in the Aceto Balsamico area where cool or temperate, sulfidic sediments with high acetate porewater concentrations and a conspicuous sulfur-rich lime-yellow surface layer were documented and sampled during previous cruises in 2008 and 2009 (Teske et al., 2016). These sediments were found in the same location and sampled again in 2016 and 2018. The resulting microbial niche is very different from the hydrothermal mixing regime and steep thermal gradients that characterize hot sediments overgrown with thick *Beggiatoaceae* mats (McKay et al., 2012, 2016).

Hydrothermal porewater signatures even persist in attenuated form in cores that, based on cold temperature and lack of microbial mats, have been categorized as background sediments when they were collected during *Alvin* dives. For example, the “background” core 4862-33 from the southern Guaymas Basin had ammonium and silicate concentrations of 300–400μM, one order of magnitude above ammonium and double the silicate concentrations for the Octopus Mound background core 4867-08. Residual hydrothermal influence is also reflected in the slightly elevated sediment temperature, 3.4°C for core 4862-33 compared to 2.9°C for all Octopus Mound cores. Based on these observations, background sediments in the strict sense (no measurable hydrothermal influence at all) may have to be collected several miles away from hydrothermal features (Teske et al., 2019).

The Octopus Mound site does not fall along the spectrum between hydrothermal sediments and background endmembers. Its uniformly cold temperatures profiles, extensive carbonate concretions, high sulfide and methane concentrations, white filamentous sulfur-oxidizing mats, chemosynthetic clams, and shallow methane hydrates are reminiscent of cold seeps at the base of the nearby Sonora Margin (Simoneit et al., 1990; Paull et al., 2007; Cruaud et al., 2017). Its *δ*^13^C-CH_4_ values are distinct from thermogenic methane in hydrothermal Guaymas Basin sediments (*δ*^13^C ≈ −38 to 43‰; McKay et al., 2016). Instead, they occupy an intermediate position between the biogenic methane at Sonora Margin seeps (*δ*^13^C ≈ −80‰; Vigneron et al., 2015) and mixed biogenic/thermogenic methane at the off-axis Ringvent site (*δ*^13^C ≈ −60 ‰; Teske et al., 2019). Interestingly, Octopus Mound methane is isotopically lighter than porewater methane from cores collected elsewhere in the Central Seep area and other off-axis seep areas of Guaymas Basin (*δ*^13^C ≈ −55 to −58‰ in MUC04; Geilert et al., 2018), and from deep subsurface sediments (*δ*^13^C ≈ −40 to −65‰; Simoneit et al., 1986), suggesting additional biogenic contributions to the porewater methane pool.

Seafloor methane undergoes microbial oxidation, as shown by the presence of methane-derived carbonates, and ANME of different types. Carbonate samples collected at the base of Octopus Mound during *Alvin* dive 4867 showed the same morphology (Figure 3) as previously dredged carbonates from this location with *δ*^13^C isotopic values of −46.6 and −44.7‰ (Geilert et al., 2018), and −45.2 to −47.6‰ (Núñez-Useche et al., 2018) that indicate the incorporation of methane-derived carbon. The sediments of Octopus Mound are populated with ANME archaea, as shown by 16S rRNA gene profiling (Figure 7B) and *mcrA* gene analysis (Figure 11). ANME-2 prefers surficial sediments with lower sulfide concentrations and tolerates some degree of oxygen exposure (Knittel et al., 2005; Rossel et al., 2011) that would be consistent with bio-irrigation by ampharetid worms, as shown by the identification of ANME-2ab archaea as the dominant ANME type in Ampharetid mats of the Hikurangi Margin seeps in New Zealand (Ruff et al., 2013). ANME-1 is generally found in reduced and sulfidic sediments (Rossel et al., 2011), consistent with the thick mat of sulfide-oxidizing *Beggiatoaceae* at the surface of the hydrate site, and with reducing conditions below the sediment surface in the “periphery” site, where bio-irrigating worms are absent.

### Controls on Microbial Community Structure

The observation that the thermally and geochemically based core categories are generally reflected in NMDS analyses of bacterial and archaeal community composition should not be taken for granted but requires an explanation. *In situ* temperatures in the upper centimeter of hydrothermally active sediments and at the mat interface – the source of the bacterial and archaeal sequences – are almost always moderate or cool, mostly at around 5°C, or averaging ~10°C when high-temperature outliers are included (McKay et al., 2012). The integrated impact of the geochemical regime within the entire sediment core – a composite of electron acceptors and donors, nutrients, and carbon sources – shapes the community composition at the sediment surface. In this view, the sediment surface may function as an integrator for microbial cells that may originate deeper within the sediment and thus reflect its average geochemical regime; these cells may move upwards with rising hydrothermal and seep fluids and accumulate at the sediment surface, as recently proposed for seep microbiota (Chakraborty et al., 2020). Consistently, microbial cell numbers (Meyer et al., 2013), cell densities of cultivable thermophiles (Teske et al., 2009), microbial lipid concentrations (Guezennec et al., 1996; Teske et al., 2002; Schouten et al., 2003), and microbial activities such as sulfate reduction rates (Weber and Jørgensen, 2002; Dowell et al., 2016) and acetate oxidation rates (Zhuang et al., 2019) are strongly focused towards maximum values within the top 3–5cm of surficial sediment. These patterns are consistent with geochemical observations that hydrothermal activity mobilizes biomass and organic carbon from the deeper sediments towards the sediment/water interface (Lin et al., 2017).

Similar to a previous study of the hydrothermal core community in Guaymas Basin sediments (Cruaud et al., 2017), sulfate-reducing and methane-oxidizing microbial groups emerge as indicator species of hydrothermal activity: the facultatively syntrophic, hydrogen-oxidizing sulfate reducer *Ca*. Desulfofervidus (Krukenberg et al., 2016), the uncultured, presumably syntrophic sulfate-reducing and alkane-oxidizing SEEP-SRB2 lineage (Krukenberg et al., 2018), and the thermophilic ANME-1 lineages (Figure 9) that are distinct from the cold-seep ANME-1a and ANME-1b groups (Biddle et al., 2012) appear characteristic for hot hydrothermal sediments, consistent with a synopsis of published 16S rRNA genes from Guaymas Basin (Dowell et al., 2016). In contrast, members of the SEEP-SRB1 lineage within the Desulfobacteraceae were present in hot hydrothermal core samples, but are more frequently found in cool or background sediment cores. Interestingly, the SEEP-SRB1 bacteria were not detected in Octopus Mound core 4867-14, although the thermal conditions would have been suitable. Methane-oxidizing syntrophic SEEP-SRB1 bacteria (Schreiber et al., 2010) could be outcompeted in this location, if bio-irrigation by mat-like assemblages of ampharetid polychaetes (Figure 3) favors aerobic methane oxidation by widespread gammaproteobacterial methylotrophs, which occur in 4867-14 and in other cores (Figure 5).

The archaeal spectrum in the diverse Guaymas Basin samples overlaps with the benthic archaeal core community of ANME-1, Thermoplasmatales (Marine Benthic Group D), Lokiarchaeota, and Bathyarchaeota that was previously proposed for Guaymas Basin hydrothermal sediments (Cruaud et al., 2017). While benthic anaerobic archaea predominate, they coexist with members of the Thaumarchaeota (*Nitrosopumilaceae*) in several hydrothermal sediments. Although aerobic, ammonia-oxidizing *Nitrosopumilaceae* (Qin et al., 2017) dominate archaeal communities in the water column, ammonium provided by hydrothermal fluids or by nitrate-reducing *Beggiatoaceae* mats enriches these archaea in surficial hydrothermal sediments as well (Winkel et al., 2014). These microbial groups with different physiologies and requirements may coexist in surficial hydrothermal sediments since this habitat provides an interface with moderate temperatures where electron donors and carbon sources from the sediment overlap with electron acceptors from the water column and create highly compressed or co-existing biogeochemical niches (Schutte et al., 2018; Buckley et al., 2019). Hydrothermal circulation in surficial sediments with microbial mats (Gundersen et al., 1992; Teske et al., 2016) can transport anaerobic or thermophilic subsurface archaea to the seawater interface and mix them with aerobic Thaumarchaeota (Qin et al., 2017). The moderate temperatures just below the sediment/seawater interface would be compatible with mesophilic microorganisms, and at the same time allow the accumulation of thermophiles and hyperthermophiles that grow at higher temperatures a few centimeters downcore (Teske et al., 2009). Since surficial hydrothermal sediments harbor diverse mesophiles as well as thermophiles, they represent an attractive target to explore the highly diverse microbiota of Guaymas Basin, with outstanding potential for evolutionary and physiological discoveries (Dombrowski et al., 2018; Seitz et al., 2019).

### Outlook: The Hydrothermal Landscape of Guaymas Basin

The contrasting hydrothermal regimes and microbial communities in Guaymas Basin reflect deeply sourced fluid and gas flow that is driven by underlying sill emplacement and local hydrothermal temperature gradients. Hydrothermal features in the southern Guaymas Basin spreading center are linked to underlying shallow sills (Lonsdale and Becker, 1985; Teske et al., 2016); the high local variability within hydrothermal areas indicates further differentiation of fluid and gas transport to the sediment surface (Ondréas et al., 2018). High-resolution bathymetries combined with shallow subbottom seismic profiles penetrating ca. 30–60m below the sediment surface show that the southern vent sites, including Mat Mound Massif (synonymous with the “Orpheus” site, Ondréas et al., 2018), Rebecca’s Roost, Cathedral Hill, and Aceto Balsamico, are linked to small sub-circular seafloor depressions with massive subsurface hydrothermal precipitate formation and lithification that creates convoluted flow paths skirting surface-breaching hydrothermal edifices. These complex and relatively shallow subsurface flow paths are consistent with frequent observations of hydrothermal hot spots and microbial mats at the base or the lower slope of these hydrothermal mounds (Dowell et al., 2016; Teske et al., 2016). The collapsed depressions are thought to facilitate the release of soluble light hydrocarbon (gas, oil, and condensates) that are transported by hydrothermal fluids towards shallow sediments where they accumulate (Ondréas et al., 2018). This setting has produced a vast patchwork of hydrothermal sediments and microbial mats, frequently visited by microbiological surveys (e.g., Teske et al., 2002; Cruaud et al., 2017; Dombrowski et al., 2018), including this study. In contrast, hydrothermal circulation at the northern sites, such as the Northern Towers area, would be linked to deeper faults and remobilize deeper hydrocarbons (Ondréas et al., 2018); we speculate that the resulting network of subsurface flow paths would be more channelized and less diversified compared to the southern area, and therefore generate large hydrothermal edifices, but fewer microbial mats and seafloor hot spots, as indicated by the *Sentry* survey of this area. Multichannel seismic surveys along the entire southern axial trough of Guaymas Basin would be needed to further substantiate such a scenario. Interestingly, a difference in subsurface hydrothermal circulation is suggested by the *δ*^13^C-CH_4_ isotopic values of hydrothermal sediments in the Northern Towers area that are slightly heavier (near −45‰, Figure 6A) than hydrothermal methane in the main sampling area of the southern axial trough (−39 to −43‰, McKay et al., 2016). This difference between the two sampling areas has been noticed previously (Teske et al., 2016), and could indicate the impact of isotopically heavier deep subsurface methane in the Northern Towers area (Simoneit et al., 1986).

The setting is entirely different at Octopus Mound. Multichannel seismic profiles show a gas pipe rising from a deeply buried sill underneath Octopus Mound that appears to funnel deeply sourced methane towards the sediment surface and into hydrate reservoirs (indicated by extensive shallow bottom simulating reflectors) around the mound (Teske et al., 2018). Deep sill-driven methane seepage is the default mechanism of hydrocarbon and methane mobilization across the spreading center and flanking regions of Guaymas Basin (Lizarralde et al., 2011). Yet, significant local variability with regard to sill depth, age, and thermal stage can also characterize the off-axis sites. For example, the deep and presumably old sill intrusion, cold seepage and predominantly biogenic *δ*^13^C-CH_4_ isotope values at Octopus Mound contrast with the shallow, recently emplaced sill, locally high temperatures and heavier, thermogenically influenced *δ*^13^C-CH_4_ isotope values at Ringvent (Teske et al., 2019), and with similar thermogenically influenced *δ*^13^C-CH_4_ values at diverse off-axis seep locations of Guaymas Basin (Geilert et al., 2018). Another factor is the localized biological modification of isotopic signatures; the Octopus Mound data indicate that biogenic methane sources are augmenting subsurface-derived methane at active seep locations. Given its compact size, habitat diversity, and geochemical contrasts, Octopus Mound and its surrounding seep sediments and shallow hydrates in the Central Seep area provide a rewarding model system for the study of sill-driven cold seepage in Guaymas Basin.

## Data Availability Statement

The 16S rRNA miseq data are deposited under Sequence Read Archive project PRJNA626075 at Genbank. The methyl coenzyme M reductase alpha subunit gene sequences are deposited at GenBank under nucleotide accession numbers MN708024 to MN708048. The geochemical data are available from the Biological and Chemical Oceanography Data Management Office at the Woods Hole Oceanographic Institution under Project Number 474317.

## Author Contributions

AT headed the R/V *Atlantis* expedition in December 2016, collected thermal profiles and samples, compiled the biological seafloor observations, developed the concept for this manuscript, and wrote the manuscript with input from all authors. Porewater geochemical analyses for ammonium, phosphate, silicate, sulfate, and sulfide were supervised and compiled by GW, and by JC for methane. DW isolated DNA from Octopus Mound samples, amplified the *mcrA* genes, and constructed the *mcrA* gene phylogeny. BM kept core records and core photographs throughout the cruise, photographed filamentous *Beggiatoaceae* from the hydrate site, and selected coring sites and recorded thermal profiles during *Alvin* dive 4869. MS and DH measured the thermal sediment gradients during dives 4866 and 4867, respectively. DH also inspected *Sentry* survey images for microbial mats and suitable sampling sites, and took *Alvin* video footage of cephalopod residents at Octopus Mound. DB and GZ measured thermal profiles during *Alvin* dives 4862 and 4871 and selected coring sites, respectively. SJ co-organized the 2016 *Atlantis* expedition. SAS co-wrote the *Sentry* proposal for this cruise, compiled the *Sentry* bathymetry, and together with DL developed the interpretation of the Octopus Mound site. SER extracted DNA from surface samples, performed 16S rRNA gene sequence-based community characterizations and statistical comparisons, and inferred phylogenetic trees.

### Conflict of Interest

The authors declare that the research was conducted in the absence of any commercial or financial relationships that could be construed as a potential conflict of interest.

## Footnotes

^1^http://4dgeo.whoi.edu/alvin

^2^https://ndsf.whoi.edu/alvin/using-alvin/sampling-equipment/

^3^https://github.com/EmilRuff/VisuaR
