# Supplementary material for: Microbial Communities Under Distinct Thermal and Geochemical Regimes in Axial and Off-Axis Sediments of Guaymas Basin
Source: Front Microbiol. 2021 Feb 12;12:633649. doi: 10.3389/fmicb.2021.633649 (PMC7906980; doi:10.3389/fmicb.2021.633649)
Supplement: Supplementary file 1 [file Data_Sheet_1.pdf]

Supplementary Material

Microbial communities under distinct thermal and geochemical regimes in axial and off-axis sediments of Guaymas Basin

Andreas Teske, Gunter Wegener, Jeffrey P. Chanton, Dylan White, Barbara MacGregor, Daniel Hoer, Dirk de Beer, Guangchao Zhuang, Matthew A. Saxton, Samantha B. Joye, Daniel Lizarralde, S. Adam Soule, S. Emil Ruff

Supplementary Figures

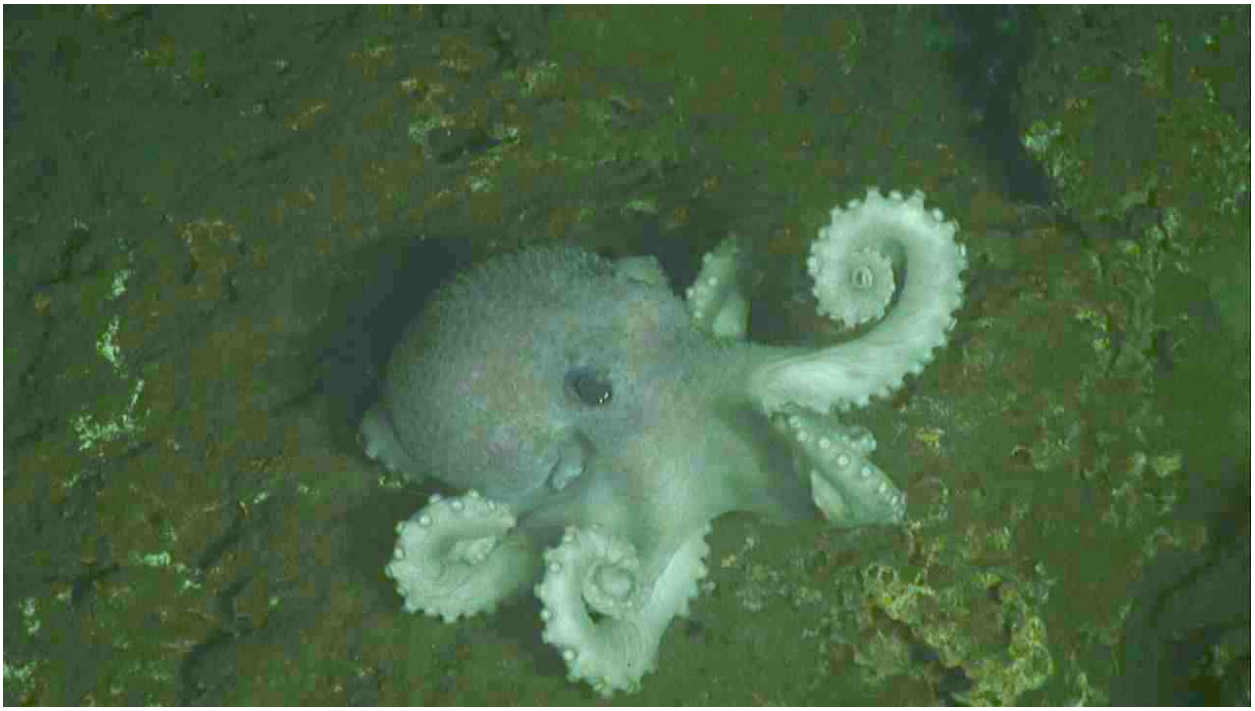

Supplementary Figure S1. Benthic Octopus at Octopus Mound. *Alvin* frame grabber screenshot, Dive 4867, GMT 20.41.57.

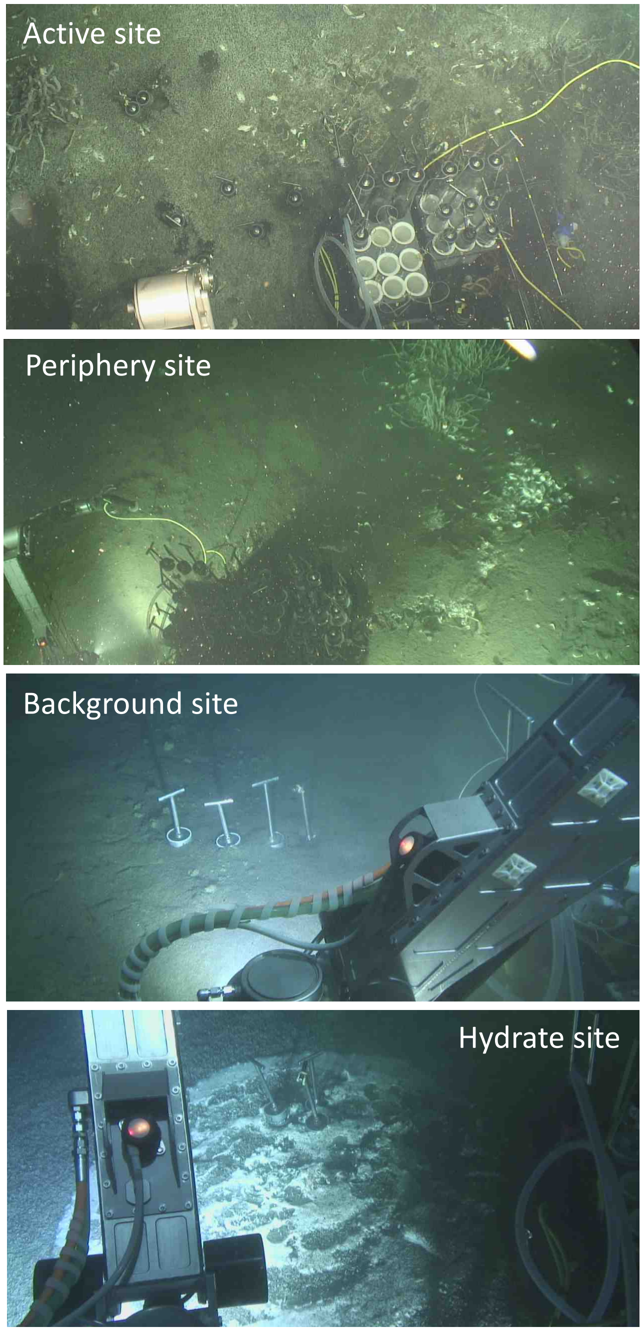

Supplementary Figure S2. *In situ* images of sampling locations at the active site, periphery site, hydrate site and background site of Octopus Mound during *Alvin* dives 4866 (active site) and 4867 (remaining sites), obtained by *Alvin* frame grabber and interior camera.

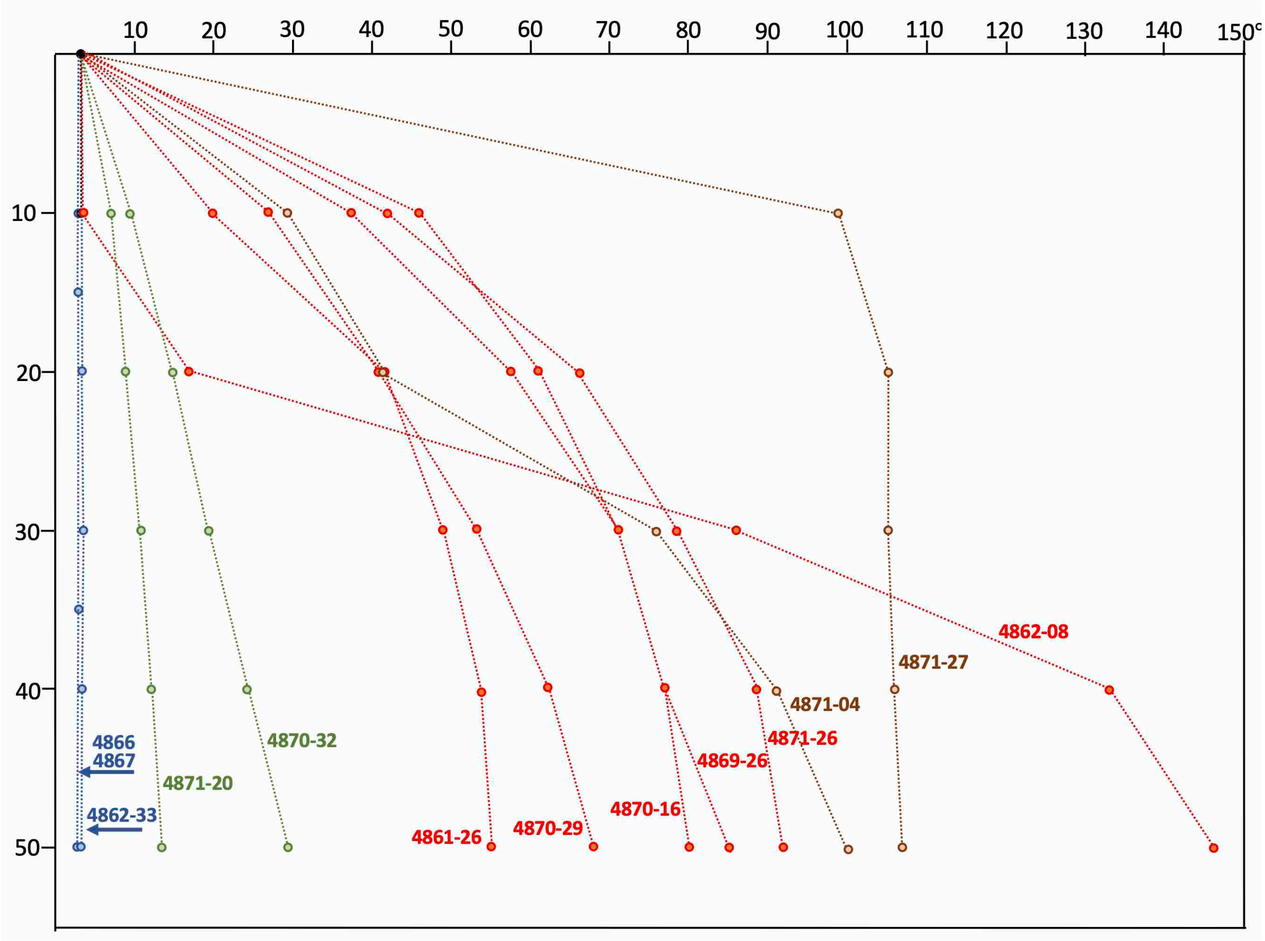

Supplementary Figure S3. Thermal profiles of background sediments (blue), temperate hydrothermal sediments (green), hot hydrothermal sediments (red), and hot hydrothermal sediments with only methane data available (brown), based on *in situ* readings of the *Alvin* heatflow probe, recorded in *Alvin* after 3-5 minutes of thermal equilibration for each measurement. Generally, thermal profiles adjacent to nearby geochemistry porewater cores are plotted (see Table 1), except for sequence data core 4861-26 where no geochemistry equivalent is currently available. For sequence data core 4870-16 and its nearby geochemistry core 4870-29, individual thermal profiles are available and both are plotted. For *Alvin* dives 4866 and 4867, thermal profiles from the Octopus Mound active site are practically indistinguishable from those at the Octopus Mound periphery, hydrate site and background site; they remain between 2.9 and 3ºC (Supplementary Table S3) and are plotted jointly as a single 4866/4867 profile.

Supplementary Table S1. Octopus Mound sampling locations for gene sequencing and porewater analyses, with *Alvin* dive and pushcore numbers. For *mcrA* gene sequencing, only sediment samples that yielded sufficient *mcrA* gene product for sequencing are listed; other sediment layers were tested but did not yield sufficient *mcrA* gene product.

	**Active site**	**Periphery site**	**Hydrate site**	**Background site**
**Fauna**	Ampharetid worm mat, *Lamellibrachia* spp., clams	no conspicuous worms or mats	Ampharetid worm mat, *Beggiatoaceae* mat	no conspicuous worms or mats
**Core numbers and features**	Grey worm tubes in top 2 cm, then dark sediment: 4866-1, 4867-14	non-reducing olive brown/ olive-green sed.: 4867-26	Dark reducing sediment, white filamentous mat on top: 4867-11	non-reducing olive brown/ olive-green sed.: 4867-8, 4867-32
**Site features**	Massive carbonate outcrops	Some meters west of the active site	Sediment buckles up; massive fractures; hydrate	seafloor sediments
**16S rRNA gene sequencing**	4867-14 (0-1 cm)	No data	No data	4867-8 (0-1 cm)
**mcrA gene sequencing**	4866-1 (0-3 cm, 9-12 cm)	4867-26 (6-9 cm)	4867-11 (0-3 cm)	Attempted but negative
**Porewater data**	4867-30	No data	No data	4867-32
**Methane conc. and $\delta^{13}C$ data**	4866-1	4867-26: No CH_4 detected	4867-11	4867-4: No CH_4 detected

Supplementary Table S2. Thermal gradients (in ºC), measured *in situ* using the 50 cm *Alvin* heatflow probe after 3-5 minutes of thermal equilibration. The gradients are plotted in Supplementary Figure S3. Thermal profiles 4871-04 and 4871-27 refer to temperatures measured after 4 minutes of thermal equilibration near these cores with methane data (Figure 6).

**core**	**4861-26**	**4862-08**	**4862-33**	**4869-26**	**4870-29**	**4870-16**	**4870-32/ 4870-02**	**4871-04**	**4871-20**	**4871-26**	**4871-27**
**10 cm**	11.4	3	3.4	37	27	46	9.5/9.1	29	6.6	42	99
**20 cm**	31.5	17	3.3	57	41	61	14.7/13.2	41	8.3	66	105
**30 cm**	42.7	86	3.8	71	53	71	19.4/17.0	76	9.8	78	105
**40 cm**	49.5	133	3.4	77	62	77	22.4/21.5	91	11.5	88	106
**50 cm**	53.0	146	3.3	85	68	80	29.2/25.3	100	12.9	92	107

Supplementary Table S3. Thermal gradients in seafloor sediments at Octopus Mound, measured during *Alvin* dives 4866 and 4867 with the 1 m heatflow probe after ca. 3-5 minutes equilibration. The probe could not be fully inserted at the hydrate site due to the presence of gas hydrate.

	**Active site, *Alvin* dives 4866 & 4867**	**Periphery site, *Alvin* dive 4867**	**Hydrate site, *Alvin* dive 4867**	**Background site, *Alvin* dive 4867**
**T1**	2.89 [water]	2.89 [water]	2.90 [water]	2.9 [interface]
**T2**	2.91 [15 cm]	2.89 [interface]	2.90 [water]	2.95 [20 cm]
**T3**	3.00 [35 cm]	2.96 [20 cm]	2.98 [10 cm]	3.03 [40 cm]
**T4**	2.99 [55 cm]	2.94 [40 cm]	3.04 [30 cm]	3.02 [60 cm]
**T5**	2.95 [75 cm]	2.90 [60 cm]	3.01 [50 cm]	3.01 [80 cm]

Supplementary Table S4. Methane $\delta^{13}C-CH_4$ values (‰VPDB) and CH_4 concentrations for *Alvin* push core samples collected during *Alvin* dives 4866, 4867 and 4871.

Alvin **samples**	**cm depth**	**$\delta^{13}C-CH_4$ (‰ VPDB)**	**St. deviation, $\delta^{13}C-CH_4$**	**CH_4 (mM)**
4866-1	0-3 cm	-69.45	0.275	1.41
4866-1	3-6 cm	-72.62	0.118	1.78
4866-1	6-9 cm	-72.04	n.d.	0.58
4866-1	9-12 cm	-74.90	0.422	0.84
4866-1	12-15 cm	-76.04	0.186	1.52
4867-11	supernatant	-68-01	0.154	4.69
4867-11	0-3 cm	-66.32	0.030	8.28
4867-11	3-6 cm	-68.64	0.060	3.62
4871-4	supernatant	-47.10	0.293	3.00
4871-4	0-3 cm	-45.18	0.107	6.27
4871-4	3-6 cm	-46.11	0.025	4.96
4871-4	6-9 cm	-46.99	0.133	8.30
4871-4	9-12 cm	-47.71	0.205	6.31
4871-4	12-15 cm	-45.76	0.109	7.75
4871-4	15-18 cm	-45.24	0.151	7.19
4871-4	18-21 cm	-45.09	0.115	6.86
4871-27	supernatant	-45.48	0.091	1.95
4871-27	0-3 cm	-39.84	0.049	4.12
4871-27	3-6 cm	-41.63	0.110	6.94
4871-27	6-9 cm	-45.04	0.265	5.64
4871-27	9-12 cm	-44.73	0.034	6.84
4871-27	12-15 cm	-45.18	0.177	6.46
4871-27	15-18 cm	-44.96	0.037	5.45
4871-27	18-21 cm	-44.57	0.102	11.55
4871-27	21-24 cm	-43.51	0.084	5.70

Supplementary Table S5. List of partial bacterial and archaeal 16S rRNA gene sequences that were used for the Methylotroph and ANME phylogenies in Figures 9 and 10**.**

Guaymas Basin Methylotroph sequences used in Phylogeny Figure 9

Guaymas_Basin_OTU_050
GATCTCTACGCATTTCACCGCTACACCTGAAATTCCACTCCCCTCTACTCAACTCTAGTTCGCCAGTTTTAA
ATGCAGTTCCCAGGTTAAGCCCGGGGCTTTCACATCTAACTTAACGAACCGCCTACGTACGCTTTACGCC
CAGTAATTCCGATTAACGCTTGCACCCTCCGTATTACCGCGGCTGCTGGCACGGAGTTAGCCGGTGCTTC
TTCTTTAGCTAATGTCAGTCTTAATGTTATTAACATTAAGGTATTCCTCACTAATGAAAGTGCTTTACAACC
CTCAGGCCTTCTTCACACACGCGGTATTGCTGGATCAGGCTTGCGCCCATTGTCCAATATTCCCCA

Guaymas_Basin_OTU_058
GATCTCTACGCATTTCACCGCTACACCTGAAATTCCACTCCCCTCTACTCAACTCTAGTTCGCCAGTTTTAA
ATGCAGTTCCCAGGTTAAGCCCGGGGCTTTCACATCTAACTTAACGAACCGCCTACGTACGCTTTACGCC
CAGTAATTCCGATTAACGCTTGCACCCTCCGTATTACCGCGGCTGCTGGCACGGAGTTAGCCGGTGCTTC
TTCTCTAGCTAATGTCAGTCTGGCATCTATTAAATTCCAGGTATTCCTCACTAGTGAAAGTGCTTTACAAC
CCTCAGGCCTTCTTCACACACGCGGTATTGCTGGATCAGGCTTGCGCCCATTGTCCAATATTCCCCA

Guaymas_Basin_OTU_068
GATCTCTACGCATTTCACTGCTACACCTGAAATTCCACTTCCCTCTCCTAAACTCTAGTTACCCAGTTTTAA
ATGCAGTTCCCAGGTTAAGCCCGGGGCTTTCACATCTAACTTAAATAACCGCCTACGAACGCTTTACGCC
CAGTAATTCCGATTAACGCTTGCACCCTCCGTATTACCGCGGCTGCTGGCACGGAGTTAGCCGGTGCTTC
TTCTAAAGGTAATGTCAAGAGACAGGATATTAACCTGCCTGTTTTCCTCCCAATTGAAAGTGCTTTACAA
CCCTCAGGCCTTCTTCACACACGCGGTATTGCTGGATCAGGCTTTCGCCCATTGTCCAATATTCCCCA

Guaymas_Basin_OTU_091
ATCTCTACGCATTTCACCGCTACACCTGAAATTCCACTCCCCTCTACTTAACTCTAGTTCGCCAGTTTTAAA
TGCAGTTCCCAGGTTAAGCCCGGGGCTTTCACATCTAACTTAACGAACCGCCTACGTACGCTTTACGCCC
AGTAATTCCGATTAACGCTTGCACCCTCCGTATTACCGCGGCTGCTGGCACGGAGTTAGCCGGTGCTTCT
TCTAAAGGTAATGTCAATCTGCCTGATATTATCTCAGCAGGTATTCCTCCCAATTGAAAGTGCTTTACAAC
CCTCAGGCCTTCTTCACACACGCGGTATTGCTGGATCAGGCTTTCGCCCATTGTCCAATATTCCCCA

Guaymas_Basin_OTU_408
ATCTCTACGCATTTCACCGCTACACCTGAAATTCCACTCTCCTCTATCCAACTCTAGTTCGCCAGTTTTAAA
TGCAGTTCCCAGGTTAAGCCCGGGGCTTTCACATCTAACTTAACGAACCGCCTACGTACGCTTTACGCCC
AGTAATTCCGATTAACGCTTGCACCCTCCGTATTACCGCGGCTGCTGGCACGGAGTTAGCCGGTGCTTCT
TCTATAGCTAATGTCAGTCTGAACGATATTATCTGCTCAGGTATTCCTCACTATTGAAAGTGCTTTACAAC
CCTCAGGCCTTCTTCACACACGCGGTATTGCTGGATCAGGCTTGCGCCCATTGTCCAATATTCCCCA

Guaymas_Basin_OTU_448

ATCTCTACGCATTTCACCGCTACACCTGAAATTCCACTCACCTCTACCATACTCTAGATATCCAGTTTCAAA
TGCAGTTCCTAGGTTGAGCCCAGGGATTTCACATCTGACTTAAATATCCGCCTACGCGCGCTTTACGCCC
AGTAATTCCGATTAACGCTTGGACCCTCCGTATTACCGCGGCTGCTGGCACGGAGTTAGCCGGTCCTTCT
TCTGACGTTAACGTCAGGGCAAACTAGTATTAATAGTCTACTTTTCCTCACGTCTGAAAGTGCTTTACAAC
CCTAAGGCCTTCTTCACACACGCGGCATTGCTGCATCAGAGTTTCCTCCATTGTGCAATATTCCCCA

Guaymas_Basin_OTU_485
GATCTCTACGCATTTCACTGCTACACCTGAAATTCCACTTCCCTCTCCTAAACTCTAGCCTTCCAGTTTTAA
ATGCAGTTCCCAGGTTAAGCCCGGGGCTTTCACATCTAACTTAAAAGGCCGCCTACGAACGCTTTACGCC
CAGTAATTCCGATTAACGCTTGCACCCTCCGTATTACCGCGGCTGCTGGCACGGAGTTAGCCGGTGCTTC
TTCTATAGGTAATGTCAAGAGACAGGATATTGACCTGCCTGTTTTCCTCCCTATTGAAAGTGCTTTACAAC
CCTCAGGCCTTCTTCACACACGCGGTATTGCTGGATCAGGCTTGCGCCCATTGTCCAATATTCCCCA

Guaymas_Basin_OTU_527
ATCTCTACGCATTTCACTGCTACACCTGAAATTCCACTTCCCTCTACCAAACTCTAGTTTGCCAGTTTTAAA
TGCAGTTCCCAGGTTGAGCCCGGGGCTTTCACATCTAACTTAACAAACCGCCTACGAACGCTTTACGCCC
AGTAATTCCGATTAACGCTTGCACCCTCCGTATTACCGCGGCTGCTGGCACGGAGTTAGCCGGTGCTTCT
TCTAAAGGTAATGTCAAGGGACGAGATATTAAGTTCGTCCGTTTTCCTCCCAATTGAAAGTGCTTTACAA
CCCTCAGGCCTTCTTCACACACGCGGTATTGCTGGATCAGGCTTTCGCCCATTGTCCAATATTCCCCA

Guaymas_Basin_OTU_537
GATCTCTACGCATTTCACCGCTACACCTGAAATTCCACTCCCCTCTACTCAACTCTAGTTCGCCAGTTTTAA
ATGCAGTTCCCAGGTTGAGCCCGGGGCTTTCACATCTAACTTAACGAACCGCCTACGTACGCTTTACGCC
CAGTAATTCCGATTAACGCTTGCACCCTCCGTATTACCGCGGCTGCTGGCACGGAGTTAGCCGGTGCTTC
TTCTATAGGTAATGTCAGTCTGTCGCATATTAAACGACAGGTATTCCTCCCTATTGAAAGTGCTTTACAAC
CCTCAGGCCTTCTTCACACACGCGGTATTGCTGGATCAGGCTTGCGCCCATTGTCCAATATTCCCCA

Guaymas Basin ANME and Methanomicrobia sequences, used in Phylogeny Figure 10

Guaymas_Basin_Arc_ASV0009
CGGCCAGGTAAGTTCCTTGGTAAATTTAGCCGCTTAACGGTTAAGCTTTCAGGGAATACTGCTTGGCTTG
GGACTGGGAGAGGTCAGAGGTACTCCGGGGGTAGGGGTGAAATCTATTAATCCCTGGGGGACCACCG
GTAGCGAAGGCGTCTGACCAGACCAGGTCCGACGGTGAGGGACGAAGGCTAGGGGCGCGAACCGGAT
TAGATACCCGGGTAGTCCTAGCTGTAAACGATGCGGGCTAGGTGTTGGCATTACTGCGAGTGGTGCCAG
TGCCGAAGGGAAGCCGTTAAGCCCGCCATCTGGGGAGTACGGTCGCAAGGCTTAAACTTAAAGGAATT
GGCGGGGGAGCACCACAACGGGTGGAGCCTGCGGTTC

Guaymas_Basin_Arc_ASV0011
CGGCTGAGTAAGTTCCTTGGGAAATTTGACCGCTTAACGGTTAAGCTATCAGGGAATACTGCTTGGCTT
GGGACCGGGAGAGGTCAGAGGTACTCCAGGGGTAGGGGTGAAATCTATTAATCCTTGGGGGACCACC
GGTAGCGAAGGCGTCTGACCAGACCGGGTCCGACGGTGAGGGACGAAGGCTGGGGTCGCGAACCGGA
TTAGATACCCGGGTAGTCCCAGCTGTAAACGATGCGGGCCAGGTGTTGGCATTACTGCGAGTGATGTCA

GTGCCAAAGGGAAGCCGTTAAGCCCGCCATCTGGGGAGTACGGTCGCAAGGCTGAAACTTAAAGAAAT
TGGCGGGGGAGCACCACAACGGGTGGAGCCTGCGGTTC

Guaymas_Basin_Arc_ASV0016
CGGCTGAGTAAGTTCCTTGGGAAATTTGACCGCTTAACGGTTAAGCTATCAGGGAATACTGCTTGGCTT
GGGACCGGGAGAGGTCAGAGGTACTCCAGGGGTAGGGGTGAAATCTATTAATCCTTGGGGGACCACC
GGTAGCGAAGGCGTCTGGCCAGACCGGGTCCGACGGTGAGGGACGAAGGCTGGGGTCGCGAACCGG
ATTAGATACCCGGGTAGTCCCAGCTGTAAACGATGCGGGCCAGGTGTTGGCATTACTGCGAGTGATGTC
AGTGCCAAAGGGAAGCCGTTAAGCCCGCCATCTGGGGAGTACGGTCGCAAGGCTGAAACTTAAAGAAA
TTGGCGGGGGAGCACCACAACGGGTGGAGCCTGCGGTTC

Guaymas_Basin_Arc_ASV0022
CGGCTGCGCAAGTTCCTTGGGAAATTTGGCCGCTTAACGGTCAACCTTTCAGGGAATACTGCGTGGCTT
GGGACCGGGAGAGGTCAGAGGTACTCTAAGGGTAGGGGTGAAATCCATTAATCCTTGGGGGACCACC
GGTAGCGAAGGCGTCTGACCAGACCGGGTCCGACGGTGAGGGACGAAGGCTAGGGGCGCGAACCGG
ATTAGATACCCGGGTAGTCCTAGCTGTAAACGATGTGGGCCAGGTGTTGGCATTACTGCGAGTGATGCC
AGTGCCGAAGGGAAGCCGTTAAGCCCACCATCTGGGGAGTACGGTCGCAAGGCTGAAACTTAAAGGAA
TTGGCGGGGGAGCACCACAACGGGTGGAGCCTGCGGTTC

Guaymas_Basin_Arc_ASV0028
CGGCTGGGTAAGTTCCTTGGGAAATTTGGCTGCTTAACAGTCAAGCTTTCAGGGAATACTGCCTGGCTT
GGGACCGGGAGAGGTCAGAGGTACTCCAGGGGTAGGGGTGAAATCCTTTAATCCTTGGGGGACCACC
GGTAGCGAAGGCGTCTGACCAGACCGGGTCCGACGGTGAGGGACGAAGGCTAGGGGCGCGAACCGG
ATTAGATACCCGGGTAGTCCTAGCAGTAAACGATGTGGGCCAGGTGTTGGCATTACTGCGAGTGATGCC
AGTGCCGAAGGGAAGCCGTTAAGCCCACCATCTGGGGAGTACGGTCGCAAGGCTGAAACTTAAAGGAA
TTGGCGGGGGAGCACCACAACGGGTGGAGCCTGCGGTTC

Guaymas_Basin_Arc_ASV0095
CAGCCAGGTAAGTTCCTTGGTAAATTTAGCCGCTTAACGGTTAAGCTTTCAGGGAATACTGCTTGGCTTG
GGACTGGGAGAGGTCAGAGGTACTCCGGGGGTAGGGGTGAAATCTATTAATCCCTGGGGGACCACCG
GTAGCGAAGGCGTCTGACCAGACCAGGTCCGACGGTGAGGGACGAAGGCTAGGGGCGCGAACCGGAT
TAGATACCCGGGTAGTCCTAGCTGTAAACGATGCGGGCTAGGTGTTGGCATTACTGCGAGTGGTGCCAG
TGCCGAAGGGAAGCCGTTAAGCCCGCCATCTGGGGAGTACGGTCGCAAGGCTTAAACTTAAAGGAATT
GGCGGGGGAGCACCACAACGGGTGGAGCCTGCGGTTC

Guaymas_Basin_Arc_ASV0105
CGGCTGCGCAAGTTCCTTGGGAAATTTGGCCGCTTAACGGTCAAGCTTTCAGGGAATACTGCGTGGCTT
GGGACCGGGAGAGGTCAGAGGTACTCTAAGGGTAGGGGTGAAATCCATTAATCCTTGGGGGACCACC
GGTAGCGAAGGCGTCTGACCAGACCGGGTCCGACGGTGAGGGACGAAGGCTAGGGGCGCGAACCGG
ATTAGATACCCGGGTAGTCCTAGCTGTAAACGATGTGGGCCAGGTGTTGGTATTACTGCGAGTGATGCC
AGTGCCGAAGGGAAGCCGTTAAGCCCACCATCTGGGGAGTACGGTCGCAAGGCTGAAACTTAAAGGAA
TTGGCGGGGGAGCACCACAACGGGTGGAGCCTGCGGTTC

Guaymas_Basin_Arc_ASV0134

CGGCCAGGTAAGTTCCTTGGTAAATTTAGCCGCTTAACGGTTAAGCTTTCAGGGAATACTGCTTGGCTTG
GGACTGGGAGAGGTCAGAGGTACTCCGGGGGTAGGGGTGAAATCTATTAATCCCTGGGGGACCACCG
GTAGCGAAGGCGTCTGACCAGACCAGGTCCGACGGTGAGGGACGAAGGCTAGGGGCGCGAACCGGAT
TAGATACCCGGGTAGTCCTAGCTGTAAACGATGCGGGCTAGGTGTTGGCATTACTGCGAGTGGTGCCAG
TGCCGAAGGGAAGCCGTTAAGCCCGCCATCTGGGGAGTACGGTCGCAAGGATTAAACTTAAAGGAATT
GGCGGGGGAGCACCACAACGGGTGGAGCCTGCGGTTC

Guaymas_Basin_Arc_ASV0138
CGGCTGGGTAAGTTCCTTGGGAAATTTGGCTGCTTAACAGTCAAGCTTTCAGGGAATACTGCCCGGCTT
GGGACCGGGAGAGGTCAGAGGTACTCCAGGGGTAGGGGTGAAATCCTTTAATCCTTGGGGGACCACC
GGTAGCGAAGGCGTCTGACCAGACCGGGTCCGACGGTGAGGGACGAAGGCTAGGGGCGCGAACCGG
ATTAGATACCCGGGTAGTCCTAGCAGTAAACGATGTGGGCCAGGTGTTGGCATTACTGCGAGTGATGCC
AGTGCCGAAGGGAAGCCGTTAAGCCCACCATCTGGGGAGTACGGTCGCAAGGCTGAAACTTAAAGGAA
TTGGCGGGGGAGCACCACAACGGGTGGAGCCTGCGGTTC

Guaymas_Basin_Arc_ASV0184
CGGCTGCGCAAGTTCCTTGGGAAATTTGGCCGCTTAACGGTCAACCTTTCAGGGAATACTGCGTGGCTT
GGGACCGGGAGAGGTCAGAGGTACTCTAAGGGTAGGGGTGAAATCCATTAATCCTTGGGGGACCACC
GGTAGCGAAGGCGTCTGACCAGACCGGGTCTGACGGTGAGGGACGAAGGCTAGGGGCGCGAACCGG
ATTAGATACCCGGGTAGTCCTAGCTGTAAACGATGTGGGCCAGGTGTTGGCATTACTGCGAGTGATGCC
AGTGCCGAAGGGAAGCCGTTAAGCCCACCATCTGGGGAGTACGGTCGCAAGGCTGAAACTTAAAGGAA
TTGGCGGGGGAGCACCACAACGGGTGGAGCCTGCGGTTC

Guaymas_Basin_Arc_ASV0252
CGGCTTGGTAAGTTCCTTGGTAAATTTAGCCGCTTAACGGTTAAGCTTTCAGGGAATACTGCTTGGCTTG
GGACTGGGAGAGGTCAGAGGTACTCCAGGGGTAGGGGTGAAATCCATTAATCCTTGGGGGACCACCG
GTAGCGAAGGCGTCTGACCAGACCAGCTCCGACGGTGAGGGACGAAGGCTAGGGGCGCGAACCGGAT
TAGATACCCGGGTAGTCCTAGCTGTAAACGATGCGGGCTAGGTGTTGGCATTACTGCGAGTGGTGCCAG
TGCCGAAGGGAAGCCGTTAAGCTCGCCATCTGGGGAGTACGGTCGCAAGGCTTAAACTTAAAGGAATT
GGCGGGGGAGCACCACAACGGGTGGAGCCTGCGGTTC

Guaymas_Basin_Arc_ASV0283
CGGCTGCGCAAGTTCCTTGGGAAATTTGGCCGCTTAACGGTCAACCTTTCAGGGAATACTGCGTGGCTT
GGGACCGGGAGAGGTCAGAGGTACTCTAAGGGTAGGGGTGAAATCCATTAATCCTTGGGGGACCACC
GGTAGCGAAGGCGTCTGACCAGACCGGGTCCGACGGTGAGGGACGAAGGCTAGGGGCGCGAACCGG
ATTAGATACCCGGGTAGTCCTAGCTGTAAACGATGTGGGCCAGGTGTTGGCATTACTGCGAGTGATGCC
AGTGCCGAAGGGAAGCCGTTAAGCCCACCATCTGGGGAGTACGGTCGCAAGGATGAAACTTAAAGGAA
TTGGCGGGGGAGCACCACAACGGGTGGAGCCTGCGGTTC

Guaymas_Basin_Arc_ASV0378
CGGCTGAGTAAGTTCCTTGGGAAATTTGACCGCTTAACGGTTAAGCTATCAGGGAATACTGCTTGGCTT
GGGACCGGGAGAGGTCAGAGGTACTTCAGGGGTAGGGGTGAAATCTATTAATCCTTGGGGGACCACC
GGTAGCGAAGGCGTCTGGCCAGACCGGGTCCGACGGTGAGGGACGAAGGCTGGGGTCGCGAACCGG
ATTAGATACCCGGGTAGTCCCAGCTGTAAACGATGCGGGCCAGGTGTTGGCATTACTGCGAGTGATGTC

AGTGCCAAAGGGAAGCCGTTAAGCCCGCCATCTGGGGAGTACGGTCGCAAGGCTGAAACTTAAAGAAA
TTGGCGGGGGAGCACCACAACGGGTGGAGCCTGCGGTTC

Guaymas_Basin_Arc_ASV0526
CGGCTGCGTAAGTTCCTTGGGAAATTTGGCCGCTTAACGGTCAAGCTTTCAGGGAATACTGCGTAGCTT
GGGACCGGGAGAGGTCAGAGGTACTCTAAGGGTAGGGGTGAAATCCATTAATCCTTGGGGGACCACC
GGTAGCGAAGGCGTCTGACCAGACCGGGTCCGACGGTGAGGGACGAAGGCTAGGGGCGCGAACCGG
ATTAGATACCCGGGTAGTCCTAGCTGTAAACGATGTGGGCCAGGTGTTGGTATTACTGCGAGTGATGCC
AGTGCCGAAGGGAAGCCGTTAAGCCCACCATCTGGGGAGTACGGTCGCAAGGCTGAAACTTAAAGGAA
TTGGCGGGGGAGCACCACAACGGGTGGAGCCTGCGGTTC

Guaymas_Basin_Arc_ASV0006
CGGCCAGGTAAGTTCTTTGGGAAATTTAACCGCTCAACGGTTAAGCTTTCAGGGAATACTGCTTGGCTTG
GGACCGGGAGAGGTCAGAGGTACTTCAAGGGTAGGGGTGAAATCCGTTAATCCTTGAGGGACCACCAG
TAGCGAAGGCGTCTGACCAGACCGGGTCCGACGGTGAGGGACGAAGGCTAGGGTCGCGAACCGGATT
AGATACCCGGGTAGTCCTAGCTGTAAACGATGCGGGCCAGGTGTTGGCATTACTGCGAGTGATGTCAGT
GCCGAAGGGAAGCCGTTAAGCCCGCCATCTGGGGAGTACGGTCGCAAGGCTGAAACTTAAAGGAATTG
GCGGGGGAGCACCACAACGGGTGGAGCCTGCGGTTC

Guaymas_Basin_Arc_ASV0073
CGGCCAGGTAAGTTCTTTGGGAAATTTAACCGCTTAACGGTTAAGCTTTCAGGGAATACTGCTTGGCTTG
GGACCGGGAGAGGTCAGAGGTACTTCAAGGGTAGGGGTGAAATCCGTTAATCCTTGAGGGACCACCAG
TAGCGAAGGCGTCTGACCAGACCGGGTCCGACGGTGAGGGACGAAGGCTAGGGTCGCGAACCGGATT
AGATACCCGGGTAGTCCTAGCTGTAAACGATGCGGGCCAGGTGTTGGCATTACTGCGAGTGATGTCAGT
GCCGAAGGGAAGCCGTTAAGCCCGCCATCTGGGGAGTACGGTCGCAAGGCTGAAACTTAAAGGAATTG
GCGGGGGAGCACCACAACGGGTGGAGCCTGCGGTTC

Guaymas_Basin_Arc_ASV0076
CAGCCAGGTAAGTTCTTTGGGAAATTTAACCGCTCAACGGTTAAGCTTTCAGGGAATACTGCTTGGCTTG
GGACCGGGAGAGGTCAGAGGTACTTCAAGGGTAGGGGTGAAATCCGTTAATCCTTGAGGGACCACCAG
TAGCGAAGGCGTCTGACCAGACCGGGTCCGACGGTGAGGGACGAAGGCTAGGGTCGCGAACCGGATT
AGATACCCGGGTAGTCCTAGCTGTAAACGATGCGGGCCAGGTGTTGGCATTACTGCGAGTGATGTCAGT
GCCGAAGGGAAGCCGTTAAGCCCGCCATCTGGGGAGTACGGTCGCAAGGCTGAAACTTAAAGGAATTG
GCGGGGGAGCACCACAACGGGTGGAGCCTGCGGTTC

Guaymas_Basin_Arc_ASV0079
CGGCTGGGTAAGTTCCTTGGGAAATTTGACCGCTTAACGGTCAAGCTATCAGGGAATACTACCCTGCTT
GGGACCGGGAGAGGTCAGAGGTACTCCAAGGGTAGGGGTGAAATCCATTAATCCTTGGGGGACCACC
GGTAGCGAAGGCGTCTGACCAGACCGGATCCGACGGTGAGGGACGAAGGCCAGGGTCGCGAACCGGA
TTAGATACCCGGGTAGTCCTGGCTGTAAACGATGCGGGCCAGGTGTTGGCATTACTGCGAGTGATGCCA
GTGCCGAAGGGAAGCCGTTAAGCCCGCCATCTGGGGAGTACGGTCGCAAGGCTGAAACTTAAAGGAAT
TGGCGGGGGAGCACCACAACGGGTGGAGCCTGCGGTTC

Guaymas_Basin_Arc_ASV0112

CGGCTGGGTAAGTTCTTTGGGAAATTTGACCGCTTAACGGTCAAGCTTTCAGGGAATACTACCTGGCTTG
GGACCGGGAGAGGTCAGAGGTACTCCAAGGGTAGGGGTGAAATCCATTAATCCTTGGGGGACCACCG
GTAGCGAAGGCGTCTGACCAGACCGGATCCGACGGTGAGGGACGAAGGCCAGGGTCGCGAACCGGAT
TAGATACCCGGGTAGTCCTGGCTGTAAACGATGCGGGCCAGGTGTTGGCATTACTGCGAGTGATGCCA
GTGCCGAAGGGAAGCCGTTAAGCCCGCCATCTGGGGAGTACGGTCGCAAGGCTGAAACTTAAAGGAAT
TGGCGGGGGAGCACCACAACGGGTGGAGCCTGCGGTTC

Guaymas_Basin_Arc_ASV0170
CGGCCAGGTAAGTTCTTTGGGAAATTTAGCCGCTCAACGGTTAAGCTTTCAGGGAATACTGCTTGGCTTG
GGACCGGGAGAGGTCAGAGGTACTTCAAGGGTAGGGGTGAAATCCGTTAATCCTTGAGGGACCACCAG
TAGCGAAGGCGTCTGACCAGACCGGGTCCGACGGTGAGGGACGAAGGCTAGGGTCGCGAACCGGATT
AGATACCCGGGTAGTCCTAGCTGTAAACGATGCGGGCCAGGTGTTGGCATTACTGCGAGTGATGTCAGT
GCCGAAGGGAAGCCGTTAAGCCCGCCATCTGGGGAGTACGGTCGCAAGGCTGAAACTTAAAGGAATTG
GCGGGGGAGCACCACAACGGGTGGAGCCTGCGGTTC

Guaymas_Basin_Arc_ASV0320
CGGCTGGGTAAGTTCTTTGGGAAATTTGACCGCTTAACGGTCAAGCTTTCAGGGAATACTACCTGGCTTG
GGACCGGGAGAGGTCAGAGGTACTCCAGGGGTAGGGGTGAAATCCATTAATCCTTGGGGGACCACCG
GTAGCGAAGGCGTCTGACCAGACCGGATCCGACGGTGAGGGACGAAGGCCAGGGTCGCGAACCGGAT
TAGATACCCGGGTAGTCCTGGCTGTAAACGATGCGGGCCAGGTGTTGGCATTACTGCGAGTGATGCCA
GTGCCGAAGGGAAGCCGTTAAGCCCGCCATCTGGGGAGTACGGTCGCAAGGCTGAAACTTAAAGGAAT
TGGCGGGGGAGCACCACAACGGGTGGAGCCTGCGGTTC

Guaymas_Basin_Arc_ASV0554
GGCCAGGTAAGTTCTTTGGGAAATTTAACCGCTCAACGGTTAAGCTTTCAGGGAATACTGCTTGGCTTG
GGACCGGGAGAGGTCAGAGGTACTTCAAGGGTAGGGGTGAAATCCGTTAATCCTTGAGGGACCACCAG
TAGCGAAGGCGTCTGACCAGACCGGGTCCGACGGTGAGGGACGAAGGCTAGGGTCGCGAACCGGATT
AGATACCCGGGTAGTCCTAGCTGTAAACGATGCGGGCCAGGTGTTGGCATTACTGCGAGTGATGTCAGT
GCCGAAGGGAAGCCGTTAAGCCCGCCATCTGGGGAGTACGGTCGCAAGGCTGAAACTTAAAGGAATTG
GCGGGGGAGCACCACAACGGGTGGAGCCTGCGGTTC

Guaymas_Basin_Arc_ASV1185
CGGCCGGGTAAGTTCTTTGGGAAATTTGACCGCTTAACGGTCAAGTTATCAGGGAATACTGCTTGGCTT
GGGACCGGGAGAGGTCAGAGGTACTCCAAGGGTAGGGGTGAAATCCGTTAATCCTTGGGGGACCACC
GGTAGCGAAGGCGTCTGACCAGACCGGATCCGACGGTGAGGGACGAAGGCTAGGGTCGCGAACCGGA
TTAGATACCCGGGTAGTCCTAGCTGTAAACGATGCGGGCCAGGTGTTGGCATTACTGCGAGTGATGTCA
GTGCCGAAGGGAAGCCGTTAAGCCCGCCATCTGGGGAGTACGGTCGCAAGGCTGAAACTTAAAGGAAT
TGGCGGGGGAGCACCACAACGGGTGGAGCCTGCGGTTC

Guaymas_Basin_Arc_ASV0002
CGGTTGAGTGAGTTCCTTGGGAAATTTGGCTGCTTAACAGTCAAACTTCCAGGGAATACTGCTCGACTTG
GGACTGGGAGAGGTCAGAGGTACTCCAGGGGTAGGGGTGAAATCCATTAATCCTTGGGGGACCACCG
GTAGCGAAGGCGTCTGACCAGACCAGGTCCGACGGTGAGGGACGAAGGCTAGGGTCGCGAACCGGAT
TAGATACCCGGGTAGTCCTAGCAGTAAACGATGCGGGCTAGGTGTTGGCATCACTGCGAGTGGTGCCA

GTGCCGAAGGGAAGCCGTTAAGCCCGCCATCTGGGGAGTACGGTCGCAAGGCTGAAACTTAAAGGAAT
TGGCGGGGGAGCACCACAACGGGTGGAGCCTGCGGTTC

Guaymas_Basin_Arc_ASV0003
CGGTTGAGTGAGTTCCTTGGGAAATTTGGCTGCTTAACAGTCAAACTTCCAGGGAATACTGCTCGACTTG
GGACTGGGAGAGGTCAGAGGTACTCCAGGGGTAGGGGTGAAATCCATTAATCCTTGGGGGACCACCG
GTAGCGAAGGCGTCTGACCAGACCAGGTCCGACGGTGAGGGACGAAGGCTAGGGTCGCGAACCGGAT
TAGATACCCGGGTAGTCCTAGCAGTAAACGATGCGGGCTAGGTGTTGGCATTACTGCGAGTGGTGCCA
GTGCCGAAGGGAAGCCGTTAAGCCCGCCATCTGGGGAGTACGGTCGCAAGGCTGAAACTTAAAGGAAT
TGGCGGGGGAGCACCACAACGGGTGGAGCCTGCGGTTC

Guaymas_Basin_Arc_ASV0004
CGGTTGAGTAAGTTCCTTGGGAAATTTGGCTGCTTAACAGTCAAGCTTCCAGGGAATACTGCTCGACTTG
GGACTGGGAGAGGTCAGAGGTACTCCAGGGGTAGGGGTGAAATCCGTTAATCCCTGGGGGACCACCG
GTAGCGAAGGCGTCTGACCAGACCAGGTCCGACGGTGAGGGACGAAGGCTAGGGTCGCGAACCGGAT
TAGATACCCGGGTAGTCCTAGCAGTAAACGATGCGGGCTAGGTGTTGGCATCACTGCGAGTGGTGCCA
GTGCCGAAGGGAAGCCGTTAAGCCCGCCATCTGGGGAGTACGGTCGCAAGGCTGAAACTTAAAGGAAT
TGGCGGGGGAGCACCACAACGGGTGGAGCCTGCGGTTC

Guaymas_Basin_Arc_ASV0014
CGGTCGGGTAAGTTCCTCGGGAAACCTCGCCGCTCAACGGTGAGGCTTCCGGGGAATACTGCCCGACTT
GGGACCGGGATGGGCCGGAGGTACTCCCGGGGTAGGGGTGAAATCCGTTGATCCCGGGAGGACCACC
CGTAGCGAAGGCGTCCGGCTGGAACGGGTCCGACGGTGAGGGACGAAGGCCAGGGGCGCGAACCGG
ATTAGATACCCGGGTAGTCCTGGCTGTAAACGATGCGGGCTTTGCGTCGGCACTGCCACGAGTGGTGCC
GGTGTTGAAGGGAAGCCGTTAAGCCCGCCGCCTGGGGAGTACGGGCGCAAGCCTGAAACTTAAAGGA
ATTGGCGGGGGAGCACTACAACGGGTGGAGCCTGCGGTTT

Guaymas_Basin_Arc_ASV0024
CGGTTGAGTAAGTTCCTTGGGAAATTTGGCTGCTTAACAGTCAAGCTTCCAGGGAATACTGCTCGACTTG
GGACTGGGAGAGGTCAGAGGTACTCCAGGGGTAGGGGTGAAATCCGTTAATCCCTGGGGGACCACCG
GTAGCGAAGGCGTCTGACCAGACCAGGTCCGACGGTGAGGGACGAAGGCTAGGGTCGCGAACCGGAT
TAGATACCCGGGTAGTCCTAGCAGTAAACGATGCGGGCTAGGTGTTGGCACTACTGCGAGTGGTGCCA
GTGCCGAAGGGAAGCCGTTAAGCCCGCCATCTGGGGAGTACGGTCGCAAGGCTGAAACTTAAAGGAAT
TGGCGGGGGAGCACCACAACGGGTGGAGCCTGCGGTTC

Guaymas_Basin_Arc_ASV0035
CGGTTGAGTAAGTTCCTTGGGAAATTTGGCTGCTTAACAGTCAAGCTTCCAGGGAATACTGCTCGACTTG
GGACTGGGAGAGGTCAGAGGTACTCCAGGGGTAGGGGTGAAATCCGTTAATCCTTGGGGGACCACCG
GTAGCGAAGGCGTCTGACCAGACCAGGTCCGACGGTGAGGGACGAAGGCTAGGGTCGCGAACCGGAT
TAGATACCCGGGTAGTCCTAGCAGTAAACGATGCGGGCTAGGTGTTGGCATCACTGCGAGTGGTGCCA
GTGCCGAAGGGAAGCCGTTAAGCCCGCCATCTGGGGAGTACGGTCGCAAGGCTGAAACTTAAAGGAAT
TGGCGGGGGAGCACCACAACGGGTGGAGCCTGCGGTTC

Guaymas_Basin_Arc_ASV0044

CAGTTGAGTAAGTTCCTTGGGAAATTTGGCTGCTTAACAGTCAAGCTTCCAGGGAATACTGCTCGACTTG
GGACTGGGAGAGGTCAGAGGTACTCCAGGGGTAGGGGTGAAATCCGTTAATCCCTGGGGGACCACCG
GTAGCGAAGGCGTCTGACCAGACCAGGTCCGACGGTGAGGGACGAAGGCTAGGGTCGCGAACCGGAT
TAGATACCCGGGTAGTCCTAGCAGTAAACGATGCGGGCTAGGTGTTGGCATCACTGCGAGTGGTGCCA
GTGCCGAAGGGAAGCCGTTAAGCCCGCCATCTGGGGAGTACGGTCGCAAGGCTGAAACTTAAAGGAAT
TGGCGGGGGAGCACCACAACGGGTGGAGCCTGCGGTTC

Guaymas_Basin_Arc_ASV0059
CGGTTGAGTAAGTTCCTTGGGAAATTTGGCTGCTTAACAGTCAAGCTTCCAGGGAATACTGCTCGACTTG
GGACTGGGAGAGGTCAGAGGTACTCCAGGGGTAGGGGTGAAATCCGTTAATCCCTGGGGGACCACCG
GTAGCGAAGGCGTCTGACCAGACCAGGTCCGACGGTGAGGGACGAAGGCTAGGGTCGCGAACCGGAT
TAGATACCCGGGTAGTCCTAGCAGTAAACGATGCGGGCTAGGTGTTGGCATCACTGCGAGTGGTGCCA
GTGCCGAAGGGAAGCCGTTAAGCCCGCCATCTGGGGAGTACGGTCGCAAGGATGAAACTTAAAGGAAT
TGGCGGGGGAGCACCACAACGGGTGGAGCCTGCGGTTC

Guaymas_Basin_Arc_ASV0075
CGGTCGGGTAAGTTCCTCGGGAAACCTCGCTGCTCAACGGTGAGGCTTCCGGGGAATACTGCCCGACTT
GGGACCGGGATGGGCCGGAGGTACTCCCGGGGTAGGGGTGAAATCCGTTGATCCCGGGAGGACCACC
CGTAGCGAAGGCGTCCGGCTGGAACGGGTCCGACGGTGAGGGACGAAGGCCAGGGGCGCGAACCGG
ATTAGATACCCGGGTAGTCCTGGCTGTAAACGATGCGGGCTTTGCGTCGGCACTGCCACGAGTGGTGCC
GGTGTTGAAGGGAAGCCGTTAAGCCCGCCGCCTGGGGAGTACGGGCGCAAGCCTGAAACTTAAAGGA
ATTGGCGGGGGAGCACTACAACGGGTGGAGCCTGCGGTTT

Guaymas_Basin_Arc_ASV0094
CGGTTGAGTAAGTTCCTTGGGAAATTTGGCTGCTTAACAGTCAAGCTTCCAGGGAATACTGCTCGACTTG
GGACTGGGAGAGGTCAGAGGTATTCCAGGGGTAGGGGTGAAATCCGTTAATCCCTGGGGGACCACCG
GTAGCGAAGGCGTCTGACCAGACCAGGTCCGACGGTGAGGGACGAAGGCTAGGGTCGCGAACCGGAT
TAGATACCCGGGTAGTCCTAGCAGTAAACGATGCGGGCTAGGTGTTGGCATCACTGCGAGTGGTGCCA
GTGCCGAAGGGAAGCCGTTAAGCCCGCCATCTGGGGAGTACGGTCGCAAGGCTGAAACTTAAAGGAAT
TGGCGGGGGAGCACCACAACGGGTGGAGCCTGCGGTTC

Guaymas_Basin_Arc_ASV0106
CGACTGAGTAAGTTCCTTGGGAAATTTGGCTGCTTAACAGTCAAGCTTCCAGGGAATACTGCTCGACTTG
GGACTGGGAGAGGTCAGAGGTACTCCAGGGGTAGGGGTGAAATCCGTTAATCCCTGGGGGACCACCG
GTAGCGAAGGCGTCTGACCAGACCAGGTCCGACGGTGAGGGACGAAGGCTAGGGTCGCGAACCGGAT
TAGATACCCGGGTAGTCCTAGCAGTAAACGATGCGGGCTAGGTGTTGGCATCACTGCGAGTGGTGCCA
GTGCCGAAGGGAAGCCGTTAAGCCCGCCATCTGGGGAGTACGGTCGCAAGGCTGAAACTTAAAGGAAT
TGGCGGGGGAGCACCACAACGGGTGGAGCCTGCGGTTC

Guaymas_Basin_Arc_ASV0125
CGGTCGGGTAAGTTCCTCGGGAAACCTCGCCGCTCAACGGTGAGGCTTCCGGGGAATACTGCCTGACTT
GGGACCGGGAGGGGCCGGAGGTACTCCCGGGGTAGGGGTGAAATCCGTTGATCCCGGGAGGACCACC
CGTAGCGAAGGCGTCCGGCTGGAACGGGTCCGACGGTGAGGGACGAAGGCCAGGGGCGCGAACCGG
ATTAGATACCCGGGTAGTCCTGGCTGTAAACGATGCGGGCTTTGCGTCGGCACTGCCACGAGTGGTGCC

GGTGTTGAAGGGAAGCCGTTAAGCCCGCCGCCTGGGGAGTACGGGCGCAAGCCTGAAACTTAAAGGA
ATTGGCGGGGGAGCACTACAACGGGTGGAGCCTGCGGTTT

Guaymas_Basin_Arc_ASV0156
CGGTTGAGTAAGTTCCTTGGGAAATTTGGCTGCTTAACAGTCAAACTTCCAGGGAATACTGCTCGACTTG
GGACTGGGAGAGGTCAGAGGTACTCCAGGGGTAGGGGTGAAATCCATTAATCCCTGGGGGACCACCG
GTAGCGAAGGCGTCTGACCAGACCAGGTCCGACGGTGAGGGACGAAGGCTAGGGTCGCGAACCGGAT
TAGATACCCGGGTAGTCCTAGCAGTAAACGATGCGGGCTAGGTGTTGGCATCACTGCGAGTGGTGCCA
GTGCCGAAGGGAAGCCGTTAAGCCCGCCATCTGGGGAGTACGGTCGCAAGGCTGAAACTTAAAGGAAT
TGGCGGGGGAGCACCACAACGGGTGGAGCCTGCGGTTC

Guaymas_Basin_Arc_ASV0179
CGGTCGGGTAAGTTCCTCGGGAAACCTCGCCGCTCAACGGTGAGGCTTCCGGGGAATACTGCCCGACTT
GGGACCGGGAGGGGCCGGAGGTACTCCCGGGGTAGGGGTGAAATCCGTTGATCCCGGGAGGACCACC
CGTAGCGAAGGCGTCCGGCTGGAACGGGTCCGACGGTGAGGGACGAAGGCCAGGGGCGCGAACCGG
ATTAGATACCCGGGTAGTCCTGGCTGTAAACGATGCGGGCTTTGCGTCGGCACTGCCACGAGTGGTGCC
GGTGTTGAAGGGAAGCCGTTAAGCCCGCCGCCTGGGGAGTACGGGCGCAAGCCTGAAACTTAAAGGA
ATTGGCGGGGGAGCACTACAACGGGTGGAGCCTGCGGTTT

Guaymas_Basin_Arc_ASV0238
CGGTCAAGTAAGTTCCTTGGGAAATTTGGCTGCTTAACAGTCAAGCTTCCAGGGAATACTGCTCGACTTG
GGACTGGGAGAGGTCAGAGGTACTCCAGGGGTAGGGGTGAAATCCGTTAATCCCTGGGGGACCACCG
GTAGCGAAGGCGTCTGACCAGACCAGGTCCGACGGTGAGGGACGAAGGCTAGGGTCGCGAACCGGAT
TAGATACCCGGGTAGTCCTAGCAGTAAACGATGCGGGCTAGGTGTTGGCACTACTGCGAGTGGTGCCA
GTGCCGAAGGGAAGCCGTTAAGCCCGCCATCTGGGGAGTACGGTCGCAAGGCTGAAACTTAAAGGAAT
TGGCGGGGGAGCACCACAACGGGTGGAGCCTGCGGTTC

Guaymas_Basin_Arc_ASV0251
CGGTCGGGTAAGTTCCTCGGGAAACCTCGCCGCTCAACGGTGAGGCTTCCGGGGAATACTGCCCGACTT
GGGACCGGGATGGGCCGGAGGTACTCCCGGGGTAGGGGTGAAATCCGTTGATCCCGGGAGGACCACC
CGTAGCGAAGGCGTCCGGCTGGAACGGGTCCGACGGTGAGGGACGAAGGCCAGGGGCGCGAACCGG
ATTAGATACCCGGGTAGTCCTGGCTGTAAACGATGCGGGCTTTGCGTCGGCACTGCCACGAGTGGTGCC
GGTGTTGAAGGGAAGCCGTTAAGCCCGCCGCCTGGGGAGTACGGGCGCAAGCATGAAACTTAAAGGA
ATTGGCGGGGGAGCACTACAACGGGTGGAGCCTGCGGTTT

Guaymas_Basin_Arc_ASV0268
CGGTTGAGTAAGTTCCTTGGGAAATTTGGCTGCTTAACAGTCAAGCTTCTAGGGAATACTGCTCGACTTG
GGACTGGGAGAGGTCAGAGGTACTCCAGGGGTAGGGGTGAAATCCGTTAATCCCTGGGGGACCACCG
GTAGCGAAGGCGTCTGACCAGACCAGGTCCGACGGTGAGGGACGAAGGCTAGGGTCGCGAACCGGAT
TAGATACCCGGGTAGTCCTAGCAGTAAACGATGCGGGCTAGGTGTTGGCATCACTGCGAGTGGTGCCA
GTGCCGAAGGGAAGCCGTTAAGCCCGCCATCTGGGGAGTACGGTCGCAAGGCTGAAACTTAAAGGAAT
TGGCGGGGGAGCACCACAACGGGTGGAGCCTGCGGTTC

Guaymas_Basin_Arc_ASV0284

CGGTCGGGTAAGTTCCTCGGGAAACCTCGCCGCTCAACGGTGAGGCTTCCGGGGAATACTGCCCGACTT
GGGACCGGGATGGGCCGGAGGTACTCCCGGGGTAGGGGTGAAATCCGTTGATCCCGGGAGGACCACC
CGTAGCGAAGGCGTCCGGCTGGAACGGGTCCGACGGTGAGGGACGAAGGCCAGGGGCGCGAACCGG
ATTAGATACCCGGGTAGTCCTGGCTGTAAACGATGCGGGCTTTGCGTCGGCACTGCCACGAGTGGTGCC
GGTGTTGAAGGGAAGCCGTTAAGCCCGCCGCCTGGGGAGTACGGGTGCAAGCCTGAAACTTAAAGGA
ATTGGCGGGGGAGCACTACAACGGGTGGAGCCTGCGGTTT

Guaymas_Basin_Arc_ASV0352
CGGTCGGGTAAGTTCCTTGGGAAATTTGGCTGCTTAACAGTCAAGCTTCCAGGGAATACTGCTCGACTT
GGGACTGGGAGAGGTCAGAGGTACTCCAGGGGTAGGGGTGAAATCCGTTAATCCCTGGGGGACCACC
GGTAGCGAAGGCGTCTGACCAGACCAGGTCCGACGGTGAGGGACGAAGGCTAGGGTCGCGAACCGGA
TTAGATACCCGGGTAGTCCTAGCAGTAAACGATGCGGGCTAGGTGTTGGCATTACTGCGAGTGGTGCCA
GTGCCGAAGGGAAGCCGTTAAGCCCGCCATCTGGGGAGTACGGTCGCAAGGCTGAAACTTAAAGGAAT
TGGCGGGGGAGCACCACAACGGGTGGAGCCTGCGGTTC

Guaymas_Basin_Arc_ASV0385
CGGTCGGGTAAATTCCTCGGGAAACCTCGCCGCTCAACGGTGAGGCTTCCGGGGAAAACTGCTCGACTT
GGGACCGGGATGGGCCGGAGGTACTCCCGGGGTAGGGGTGAAATCCGTTGATCCCGGGAGGACCACC
CGTAGCGAAGGCGTCCGGCTGGAACGGGTCCGACGGTGAGGGACGAAGGCCAGGGGCGCGAACCGG
ATTAGATACCCGGGTAGTCCTGGCTGTAAACGATGCGGGCTTTGCGTCGGCACCGCCACGAGTGGTGCC
GGTGTTGAAGGGAAGCCGTTAAGCCCGCCGCCTGGGGAGTACGCCCGCAAGGGTGAAACTTAAACCAA
TTGGCGGGGGAGCACTACAACGGGTGGAGCCTGCGGTTT

Guaymas_Basin_Arc_ASV0414
CGGTTGAGTAAGTTCCTTGGGAAATTTGGCTGCTTAACAGTCAAACTTCCAGGGAATACTGCTCGACTTG
GGACCGGGAGAGGTCAGAGGTACCTCAAGGGTAGGGGTGAAATCCGTTAATCCTTGGGGGACCACCA
GTAGCGAAGGCGTCTGACCAGACCGGATCCGACGGTGAGGGACGAAGGCTAGGGTAGCGAACCGGAT
TAGATACCCGGGTAGTCCTAGCTGTAAACGATGCGGGCTAGGTGTTGGCACTACTGCGAGTGGTGCCA
GTGCCGAAGGGAAGCCGTTAAGCCCGCCACCTGGGGAGTACGGTCGCAAGGCTGAAACTTAAAGGAAT
TGGCGGGGGAGCACCACAACGGGTGGAGCCTGCGGTTC

Guaymas_Basin_Arc_ASV0224
CTGTTTAATAAGTCCTTTGGGAAATCTGGCAGCTTAACTGTCAGGCTGCTAAAGGATACTGTTAAACTTG
GGACCGGTAGACGTAGGGGGTACTCCAGGGGTAGGAGTGAAATCTTGTAATCCCTGGGGGACCATCTG
TGGCGAAGGCGCCCTACGAGAACGGGTCCGACGGTGAGGGACGAAAGCTAGGGGAGCAAACCGGATT
AGATACCCGGGTAGTCCTAGCTGTAAACGATGCTCGCTAGGTGTCAGGCACGGTGCGTCCGTGTCTGGT
GCCGCAGGGAAGCCGTTAAGCGAGCCACCTGGGGAGTACGGTCGCAAGGCTGAAACTTAAAGGAATT
GGCGGGGGAGCACTACAACGGGTGGAGCCTGCGGTTT

Guaymas_Basin_Arc_ASV0442
CTGTTTAATAAGTCCTTTGGGAAATCTGGCAGCTTAACTGTCAGGCTGCTAAAGGATACTGTTAAACTTG
GGACCGGGAGACGTAGGGGGTACTCCAGGGGTAGGAGTGAAATCTTGTAATCCCTGGGGGACCATCTG
TGGCGAAGGCGCCCTACGAGAACGGGTCCGACGGTGAGGGACGAAAGCTAGGGGAGCAAACCGGATT
AGATACCCGGGTAGTCCTAGCTGTAAACGATGCTCGCTAGGTGTCCGGCACGGTGCGTCCGTGTCTGGT

GCCGCAGGGAAGCCGTTAAGCGAGCCACCTGGGAAGTACGGTCGCAAGGCTGAAACTTAAAGGAATTG
GCGGGGGAGCACTACAACGGGTGGAGCCTGCGGTTT

Guaymas_Basin_Arc_ASV1442
CGGCTTAATAAGTCCTTTGGGAAATCTGGCAGCTTAACTGTCAGGCTGCTAAAGGATACTGTTAAGCTTG
GGACCGGGAGAGGTAGGGGGTACTCCAGGGGTAGGAGTGAAATCTTGTAATCCCTGGGGGACCATCT
GTGGCGAAGGCGCCCTACCAGAACGGGTCCGACGGTGAGGGACGAAAGCTAGGGGAGCAAACCGGAT
TAGATACCCGGGTAGTCCTAGCTGTAAACGATGCTCGCTAGGTGTCAGGTACGGTGCGTCCGTGTCTGG
TGCCGCAGGGAAGCCGCTAAGCGAGCCACCTGGGAAGTACGGTCGCAAGGCTGAAACTTAAAGGAATT
GGCGGGGGAGCACTACAACGGGTGGAGCCTGCGGTTT

Guaymas_Basin_Arc_ASV0017
CGGCCTGATAAGTCCTTTGGGAAATCTGGCAGCTTAACTGTCAGGCTTCTAAGGGATACTGTCAGGCTT
GGGACCGGAAGAGGTAAGGGGTACTCCAGGGGTAGGAGTGAAATCTTGTAATCCCTGGGGGACCATCT
GTGGCGAAGGCGCCTTACCAGAACGGGTCCGACGGTGAGGGACGAAAGCTAGGGGAGCAAACCGGAT
TAGATACCCGGGTAGTCCTAGCTGTAAACGATGCTCGCTAGGTGTCAGACACGGTGCGACCGTGTTTGG
TGCCGCAGGGAAGCCGTTAAGCGAGCCACCTGGGAAGTACGGTCGCAAGGCTGAAACTTAAAGGAATT
GGCGGGGGAGCACTACAACGGGTGGAGCCTGCGGTTT

Guaymas_Basin_Arc_ASV0041
CGGCCTGATAAGTCCTTTGGGAAATCTGACAGCTTAACTGTCAGGCTTCTAAGGGATACTGTCAGGCTTG
GGACCGGAAGAGGTAAGGGGTACTCCAGGGGTAGGAGTGAAATCTTGTAATCCCTGGGGGACCATCTG
TGGCGAAGGCGCCTTACCAGAACGGGTCCGACGGTGAGGGACGAAAGCTAGGGGAGCAAACCGGATT
AGATACCCGGGTAGTCCTAGCTGTAAACGATGCTCGCTAGGTGTCAGACACGGTGCGACCGTGTTTGGT
GCCGCAGGGAAGCCGTTAAGCGAGCCACCTGGGAAGTACGGTCGCAAGGCTGAAACTTAAAGGAATTG
GCGGGGGAGCACTACAACGGGTGGAGCCTGCGGTTT

Guaymas_Basin_Arc_ASV0089
CGGCTTGATAAGTCTTTTGGGAAATCTGGCAGCTTAACTGTCAGGCTTCTAAGGGATACTGTCAGGCTTG
GGACCGGAAGAGGTAAGGGGTACTCCAGGGGTAGGAGTGAAATCTTGTAATCCCTGGGGGACCATCTG
TGGCGAAGGCGCCTTACCAGAACGGGTCCGACGGTGAGGGACGAAAGCTAGGGGAGCAAACCGGATT
AGATACCCGGGTAGTCCTAGCTGTAAACGATGCTCGCTAGGTGTCAGACACGGTGCGACCGTGTTTGGT
GCCGCAGGGAAGCCGTTAAGCGAGCCACCTGGGAAGTACGGTCGCAAGGCTGAAACTTAAAGGAATTG
GCGGGGGAGCACTACAACGGGTGGAGCCTGCGGTTT

Guaymas_Basin_Arc_ASV0162
CGGCTTGATAAGTCCTTTGGGAAATCTGGCAGCTTAACTGTCAGGCTTCTAAGGGATACTGTCAGGCTTG
GGACCGGAAGAGGTAAGGGGTACTCCAGGGGTAGGAGTGAAATCTTGTAATCCCTGGGGGACCATCTG
TGGCGAAGGCGCCTTACCAGAACGGGTCCGACGGTGAGGGACGAAAGCTAGGGGAGCAAACCGGATT
AGATACCCGGGTAGTCCTAGCTGTAAACGATGCTCGCTAGGTGTCAGACACGGTGCGACCGTGTTTGGT
GCCGCAGGGAAGCCGTTAAGCGAGCCACCTGGGAAGTACGGTCGCAAGGCTGAAACTTAAAGGAATTG
GCGGGGGAGCACTACAACGGGTGGAGCCTGCGGTTT

Guaymas_Basin_Arc_ASV0228

CGGCCTGATCAGTCCTTTGGGAAATCTGGCAGCTTAACTGTCAGGCTTCTAAGGGATACTGTCAGGCTTG
GGACCGGAAGAGGTAAGGGGTACTCCAGGGGTAGGAGTGAAATCTTGTAATCCCTGGGGGACCATCTG
TGGCGAAGGCGCCTTACCAGAACGGGTCCGACGGTGAGGGACGAAAGCTAGGGGAGCAAACCGGATT
AGATACCCGGGTAGTCCTAGCTGTAAACGATGCTCGCTAGGTGTCAGACACGGTGCGACCGTGTTTGGT
GCCGCAGGGAAGCCGTTAAGCGAGCCACCTGGGAAGTACGGTCGCAAGGCTGAAACTTAAAGGAATTG
GCGGGGGAGCACTACAACGGGTGGAGCCTGCGGTTT

Guaymas_Basin_Arc_ASV0332
CGGCTTGATAAGTCTTTTGGGAAATCTGGCAGCTTAACTGTCAGGCTTCTAAGAGATACTGTCAGGCTTG
GGACCGGAAGAGGTAAGGGGTACTCCAGGGGTAGGAGTGAAATCTTGTAATCCCTGGGGGACCATCTG
TGGCGAAGGCGCCTTACCAGAACGGGTCCGACGGTGAGGGACGAAAGCTAGGGGAGCAAACCGGATT
AGATACCCGGGTAGTCCTAGCTGTAAACGATGCTCGCTAGGTGTCAGACACGGTGCGACCGTGTTTGGT
GCCGCAGGGAAGCCGTTAAGCGAGCCACCTGGGAAGTACGGTCGCAAGGCTGAAACTTAAAGGAATTG
GCGGGGGAGCACTACAACGGGTGGAGCCTGCGGTTT

Guaymas_Basin_Arc_ASV0851
CGGCCTGAAAAGTCCTTTGGGAAATCTGACAGCTTAACTGTCAGGCTTCTAAGGGATACTGTCAGGCTT
GGGACCGGGAGAGGTAAGGGGTACTCAAAGGGTAGGAGTGAAATCTTGTAATCCTTTGGGGACCATCT
GTGGCGAAGGCGCCTTACCAGAACGGGTCCGACGGTGAGGGACGAAAGCTAGGGGAGCAAACCGGAT
TAGATACCCGGGTAGTCCTAGCCGTAAACGATGCTCGCTAGGTGTCTGGCACGGTGCGTCCGTGTCCGG
TGCCGCAGGGAAGCCGTTAAGCGAGCCACCTGGGAAGTACGGTCGCAAGGCTGAAACTTAAAGGAATT
GGCGGGGGAGCACTACAACGGGTGGAGCCTGCGGTTT

Guaymas_Basin_Arc_ASV0900
CGGCTTGATAAGTCCTTTGGGAAATCTGGTAGCTTAACTATCAGGCTTCTAAGGGATACTGTCAGGCTTG
GGACCGGGAGAGGTAAGGGGTACTCCAGGGGTAGGAGTGAAATCTTGTAATCCCTGGGGGACCATCT
GTGGCGAAGGCGCCTTACCAGAACGGGTCCGACGGTGAGGGACGAAAGCTAGGGGAGCAAACCGGAT
TAGATACCCGGGTAGTCCTAGCTGTAAACGATGCTCGCTAGGTGTCAGGTACGGTGCGACCGTGTCTGG
TGCCGCAGGGAAGCCGTTAAGCGAGCCACCTGGGAAGTACGGTCGCAAGGCTGAAACTTAAAGGAATT
GGCGGGGGAGCACTACAACGGGTGGAGCCTGCGGTTT

Guaymas_Basin_Arc_ASV1041
CGGCTTGAACAGTCTTTTGGGAAATCTGACAGCTTAACTGTTAGGCTTCTAAGGGATACTATCAGGCTTG
GGACCGGGAGAGGTAAGGGGTACTCCAAGGGTAGGAGTGAAATCTTGTAATCCTTGGGGGACCATCTG
TGGCGAAGGCGCCTTACCAGAACGGGTCCGACGGTGAGGGACGAAAGCTAGGGGAGCAAACCGGATT
AGATACCCGGGTAGTCCTAGCTGTAAACGATGCTCGCTAGGTGTCAGGTACGGTGCGACCGTGTCTGGT
GCCGCAGGGAAGCCGTTAAGCGAGCCACCTGGGAAGTACGGTCGCAAGGCTGAAACTTAAAGGAATTG
GCGGGGGAGCACTACAACGGGTGGAGCCTGCGGTTT

Guaymas_Basin_Arc_ASV0001
CGGTTCGATAAGTCCTCTGGGAAATCTGGTTGCTTAACAATCAGACTGCCAAGGGATACTGTCGAACTT
GAGACCGGGAGAGGTAAGAGGTACTTCAGGGGTAGGAGTGAAATCTTGTAATCCCTGGGGGACCATCT
GTGGCGAAGGCGTCTTACCAGAACGGGTCTGACGGTGAGGGACGAAAGCTGGGGGCGCAAACCGGAT
TAGATACCCGGGTAGTCCCAGCCGTAAACGATGCTCGCTATGTGTCAGGTACGGTGTGACCGTATCTGG

TGCCGTAGGGAAGCCGTGAAGCGAGCCACCTGGGAAGTACGGTCGCAAGACTGAAACTTAAAGGAATT
GGCGGGGGAGCACTACAACGGGTGGAGCCTGCGGTTT

Guaymas_Basin_Arc_ASV0005
CGGTTCGATAAGTCCTCTGGGAAATCTGGTTGCTTAACAATCAGACTGCCAAGGGATACTGTCGAACTT
GAGACCGGGAGAGGTAAGAGGTACTTCAGGGGTAGGAGTGAAATCTTGTAATCCCTGGGGGACCATCT
GTGGCGAAGGCGTCTTACCAGAACGGGTCTGACGGTGAGGGACGAAAGCTGGGGGCGCAAACCGGAT
TAGATACCCGGGTAGTCCCAGCCGTAAACGATGCTCGCTATGTGTCAGGTACGGTGTGACCGTATCTGG
TGCCGTAGGGAAGCCGTGAAGCGAGCCACCTGGGAAGTACGGCCGCAAGGCTGAAACTTAAAGGAATT
GGCGGGGGAGCACTACAACGGGTGGAGCCTGCGGTTT

Guaymas_Basin_Arc_ASV0013
CAGTTCGATAAGTCCTCTGGGAAATCTGGTTGCTTAACAATCAGACTGCCAAGGGATACTGTCGAACTTG
AGACCGGGAGAGGTAAGAGGTACTTCAGGGGTAGGAGTGAAATCTTGTAATCCCTGGGGGACCATCTG
TGGCGAAGGCGTCTTACCAGAACGGGTCTGACGGTGAGGGACGAAAGCTGGGGGCGCAAACCGGATT
AGATACCCGGGTAGTCCCAGCCGTAAACGATGCTCGCTATGTGTCAGGTACGGTGTGACCGTATCTGGT
GCCGTAGGGAAGCCGTGAAGCGAGCCACCTGGGAAGTACGGTCGCAAGACTGAAACTTAAAGGAATTG
GCGGGGGAGCACTACAACGGGTGGAGCCTGCGGTTT

Guaymas_Basin_Arc_ASV0021
CGGTTCGATAAGTCCTCTGGGAAATCTGGTTGCTTAACAATCAGACTGCCAAGGGATACTGTCGAACTT
GAGACCGGGAGAGGTAAGAGGTACTTCAGGGGTAGGAGTGAAATCTTGTAATCCCTGGGGGACCATCT
GTGGCGAAGGCGTCTTACCAGAACGGGTCTGACGGTGAGGGACGAAAGCTGGGGGCGCAAACCGGAT
TAGATACCCGGGTAGTCCCAGCCGTAAACGATGCTCGCTATGTGTCAGGTACGGTGTGACCGTATCTGG
TGCCGTAGGGAAGCCGTGAAGCGAGCCACCTGGGAAGTACGGTCGCAAGAATGAAACTTAAAGGAATT
GGCGGGGGAGCACTACAACGGGTGGAGCCTGCGGTTT

Guaymas_Basin_Arc_ASV0026
CGGTTTGGTAAGTCCTCTGGAAAATCTGGTTGCTCAACAATCAGACTGCCAAGGGATACTGTCGAACTT
GAGACCGGGAGAGGTAAGAGGTACTTCAGGGGTAGGAGTGAAATCTTGTAATCCCTGGGGGACCATCT
GTGGCGAAGGCGTCTTACCAGAACGGGTCTGACGGTGAGGGACGAAAGCTGGGGGCGCGAACCGGAT
TAGATACCCGGGTAGTCCCAGCCGTAAACGATGCTCGCTATGTGTCAGGTACGGTGCGACCGTATCTGG
TGCCGTAGGGAAGCCGTGAAGCGAGCCACCTGGGAAGTACGGCCGCAAGGCTGAAACTTAAAGGAATT
GGCGGGGGAGCACTACAACGGGTGGAGCCTGCGGTTT

Guaymas_Basin_Arc_ASV0030
CGACTCGATAAGTCCTCTGGGAAATCTGGTTGCTTAACAATCAGACTGCCAAGGGATACTGTCGAACTT
GAGACCGGGAGAGGTAAGAGGTACTTCAGGGGTAGGAGTGAAATCTTGTAATCCCTGGGGGACCATCT
GTGGCGAAGGCGTCTTACCAGAACGGGTCTGACGGTGAGGGACGAAAGCTGGGGGCGCAAACCGGAT
TAGATACCCGGGTAGTCCCAGCCGTAAACGATGCTCGCTATGTGTCAGGTACGGTGTGACCGTATCTGG
TGCCGTAGGGAAGCCGTGAAGCGAGCCACCTGGGAAGTACGGTCGCAAGACTGAAACTTAAAGGAATT
GGCGGGGGAGCACTACAACGGGTGGAGCCTGCGGTTT

Guaymas_Basin_Arc_ASV0037

CGGTTCGGTAAGTCTTTTGGGAAATCTGGTTGCTTAACAATCAGACTGCCAAGGGATACTGCTGAACTTG
AGACCGGGAGAGGTAAGAGGTACTTCAGGGGTAGGAGTGAAATCTTGTGATCCCTGGGGGACCATCTG
TGGCGAAGGCGTCTTACCAGAACGGGTCTGACGGTGAGGGACGAAAGCTGGGGGCGCAAACCGGATT
AGATACCCGGGTAGTCCCAGCCGTAAACGATGCTCGCTATGTGTCAGGTACGGTGCGACCGTATCTGGT
GCCGTAGGGAAGCCGTGAAGCGAGCCACCTGGGAAGTACGGTCGCAAGACTGAAACTTAAAGGAATTG
GCGGGGGAGCACTACAACGGGTGGAGCCTGCGGTTT

Guaymas_Basin_Arc_ASV0077
CGGTTTGGTAAGTCCTCTGGAAAATCTGGTTGCTCAACAATCAGACTGCCAAGGGATACTGTCAAACTTG
AGACCGGGAGAGGTAAGAGGTACTTCAGGGGTAGGAGTGAAATCTTGTAATCCCTGGGGGACCATCTG
TGGCGAAGGCGTCTTACCAGAACGGGTCTGACGGTGAGGGACGAAAGCTGGGGGCGCGAACCGGATT
AGATACCCGGGTAGTCCCAGCCGTAAACGATGCTCGCTATGTGTCAGGTACGGTGCGACCGTATCTGGT
GCCGTAGGGAAGCCGTGAAGCGAGCCACCTGGGAAGTACGGCCGCAAGGCTGAAACTTAAAGGAATT
GGCGGGGGAGCACTACAACGGGTGGAGCCTGCGGTTT

Guaymas_Basin_Arc_ASV0128
CGGTTCGGTAAGTCCTCTGGAAAATCTGGTTGCTCAACAATCAGACTGCCAAGGGATACTGTCGAACTT
GAGACCGGGAGAGGTAAGAGGTACTTCAGGGGTAGGAGTGAAATCTTGTAATCCCTGGGGGACCATCT
GTGGCGAAGGCGTCTTACCAGAACGGGTCTGACGGTGAGGGACGAAAGCTGGGGGCGCGAACCGGAT
TAGATACCCGGGTAGTCCCAGCCGTAAACGATGCTCGCTATGTGTCAGGTACGGTGCGACCGTATCTGG
TGCCGTAGGGAAGCCGTGAAGCGAGCCACCTGGGAAGTACGGCCGCAAGGCTGAAACTTAAAGGAATT
GGCGGGGGAGCACTACAACGGGTGGAGCCTGCGGTTT

Guaymas_Basin_Arc_ASV0148
CGGTTCGGTAAGTCCTTTGGGAAATCTGGTTGCTTAACAATCAGACTGCCAAGGGATACTGCTGAACTT
GAGACCGGGAGAGGTAAGAGGTACTTCAGGGGTAGGAGTGAAATCTTGTGATCCCTGGGGGACCATCT
GTGGCGAAGGCGTCTTACCAGAACGGGTCTGACGGTGAGGGACGAAAGCTGGGGGCGCAAACCGGAT
TAGATACCCGGGTAGTCCCAGCCGTAAACGATGCTCGCTATGTGTCAGGTACGGTGCGACCGTATCTGG
TGCCGTAGGGAAGCCGTGAAGCGAGCCACCTGGGAAGTACGGTCGCAAGACTGAAACTTAAAGGAATT
GGCGGGGGAGCACTACAACGGGTGGAGCCTGCGGTTT

Guaymas_Basin_Arc_ASV0292
CGGTTTGATAAGTCTTCTGGGAAATCTGGTTGCTTAACAATCAGACTGCCAAGAGATACTGTCAAACTTG
AGACCGGGAGAGGTAAGAGGTACTTCAGGGGTAGGAGTGAAATCTTGTAATCCCTGGGGGACCATCTG
TGGCGAAGGCGTCTTACCAGAACGGGTCTGACGGTGAGGGACGAAAGCTGGGGGCGCAAACCGGATT
AGATACCCGGGTAGTCCCAGCCGTAAACGATGCTCGCTATGTGTCAGGTACGGTGTGACCGTATCTGGT
GCCGTAGGGAAGCCGTGAAGCGAGCCACCTGGGAAGTACGGCCGCAAGGCTGAAACTTAAAGGAATT
GGCGGGGGAGCACTACAACGGGTGGAGCCTGCGGTTT

Guaymas_Basin_Arc_ASV0469
CGGTTTGGTAAGTCCTCTGGAAAATCTGGTTGCTCAACAATCAGACTGCCAAGGGATACTGTCAAACTTG
AGACCGGGAGAGGTAAGAGGTACTTCAGGGGTAGGAGTGAAATCTTGTAATCCCTGGGGGACCATCTG
TGGCGAAGGCGTCTTACCAGAACGGGTCTGACGGTGAGGGACGAAAGCTGGGGGCGCGAACCGGATT
AGATACCCGGGTAGTCCCAGCCGTAAACGATGCTCGCTATGTGTCAGGTACGGTGCGACCATATCTGGT

GCCGTAGGGAAGCCGTGAAGCGAGCCACCTGGGAAGTACGGCCGCAAGGCTGAAACTTAAAGGAATT
GGCGGGGGAGCACTACAACGGGTGGAGCCTGCGGTTT

Guaymas_Basin_Arc_ASV0567
CGACCTGGTAAGTCCTCTGGAAAATCTGGTTGCTCAACAATCAGACTGCCAAGGGATACTGTCGAACTT
GAGACCGGGAGAGGTAAGAGGTACTTCAGGGGTAGGAGTGAAATCTTGTAATCCCTGGGGGACCATCT
GTGGCGAAGGCGTCTTACCAGAACGGGTCTGACGGTGAGGGACGAAAGCTGGGGGCGCGAACCGGAT
TAGATACCCGGGTAGTCCCAGCCGTAAACGATGCTCGCTATGTGTCAGGTACGGTGCGACCGTATCTGG
TGCCGTAGGGAAGCCGTGAAGCGAGCCACCTGGGAAGTACGGCCGCAAGGCTGAAACTTAAAGGAATT
GGCGGGGGAGCACTACAACGGGTGGAGCCTGCGGTTT

Guaymas_Basin_Arc_ASV0911
CGGTTTGATAAGTCTTCTGGGAAATCTGATTGCTCAACAATCAGGCTGCCAAGGGATACTGTCAAACTTG
AGACCGGGAGAGGTAAGAGGTACTTCAGGGGTAGGAGTGAAATCTTATAATCCTTGGGGGACCGTCTG
TGGCGAAGGCGTCTTACCAGAACGGGTCTGACGGTGAGGGACGAAAGCTGGGGGCGCGAACCGGATT
AGATACCCGGGTAGTCCCAGCCGTAAACGATGCTCGCTATGTGTCAGATACGGTGCGACCGTATCTGGT
GCCGTAGGGAAGCCGTGAAGCGAGCCACCTGGGAAGTACGGCCGCAAGGCTGAAACTTAAAGGAATT
GGCGGGGGAGCACTACAACGGGTGGAGCCTGCGGTTT

Guaymas_Basin_Arc_ASV0988
CGGTTCGGTAAGTCCTTTGGGAAATCTGGTTGCTTAACAATTAGGCTGCTAAGGGATACTGTCGAACTTG
AGACCGGGAGAGGTAAGAGGTACTTCAGGGGTAGGAGTGAAATCTTGTAATCCTTGGGGGACCATCTG
TGGCGAAGGCGTCTTACTAGAACGGGTCTGACGGTGAGGGGCGAAAGCTGGGGGCGCGAACCGGATT
AGATACCCGGGTAGTCCCAGCCGTAAACGATGCTCGCTATGTGTCAGGTACGGTGTGACCGTATCTGGT
GCCGTAGGGAAGCCGTGAAGCGAGCCACCTGGGAAGTACGGCCGCAAGGCTGAAACTTAAAGGAATT
GGCGGGGGAGCACTACAATGGGTGGAGCCTGCGGTTT

Guaymas_Basin_Arc_ASV1283
CGGTTTGATAAGTCCTCTGGGAAATCTGATTGCTCAACAATCAGGCTGCCAAGGGATACTGTCAAACTTG
AGACCGGGAGAGGTAAGAGGTACTTCAAGGGTAGGAGTGAAATCTTATAATCCTTGGGGGACCGTCTG
TGGCGAAGGCGTCTTACCAGAACGGGTCTGACGGTGAGGGACGAAAGCTGGGGGCGCGAACCGGATT
AGATACCCGGGTAGTCCCAGCCGTAAACGATGCTCGCTATGTGTCAGGTACGGTGCGACCGTATCTGGT
GCCGTAGGGAAGCCGTGAAGCGAGCCACCTGGGAAGTACGGCCGCAAGGCTGAAACTTAAAGGAATT
GGCGGGGGAGCACTACAACGGGTGGAGCCTGCGGTTT

Guaymas_Basin_Arc_ASV0443
CGGTTTAGTAAGTTTCTTAGGAAATCTAGCAGCTTAACTGTTAGACGTCTAAGAGATACTGCTATGACTT
GGGACCGGGAGAGGTAGGAGGTACTCCAGGGGTAGGGGTGAAATCTTGTAATCCCTGGGGGACCATC
GATGGCGAAGGCATCCTACCAGAACGGGTTCGACGGTGAGGGACGAAAGCTGGGGGCACAAACCGGA
TTAGATACCCGGGTAGTCCCAGCTGTAAACGATGCTCGCTAGGTGTCAGATACGGTGCGTCCGTATTTG
GTGCCGTAGGGAAACCGTGAAGCGAGCCGCCTGGGAAGTACGGTCGCAAGGCTGAAACTTAAAGGAA
TTGGCGGGGGAGCACTACAACGGGTGGAGCCTGCGGTTC

Guaymas_Basin_Arc_ASV1417

CGGTTTGGTAAGTCTCTTAGGAAATCTGGCAGCTTAACTGTTAGGCGTCTAAGAGATACTGCCAAACTTG
GGATCGGGAGAGGTAGGAGGTACTCCAGGGGTAGGGGTGAAATCTTGTAATCCTTGGAGGACCATCG
ATGGCGAAGGCATCCTACCAGAACGAGTCCGACGGTGAGGGACGAAAGCTGGGGGCACAAACCGGAT
TAGATACCCGGGTAGTCCCAGCTGTAAACGATGCTCGCTAGGTGTCAGATACGGTGCGTCCGTATTTGG
TGCCGTAGGGAAACCGTGAAGCGAGCCGCCTGGGAAGTACGGTCGCAAGGCTGAAACTTAAAGGAATT
GGCGGGGGAGCACTACAACGGGTGGAGCCTGCGGTTC

Guaymas_Basin_Arc_ASV0149
CGGTTTGATCAGTCTTCCGGGAAATCTGACAGCTCAACTGTTAGGCTTCCGGTGGATACTGTCAGACTTG
GGACCGGGAGAGGTAAGAGGTACTACAGGGGTAGGAGTGAAATCTTGTAATCCCTGTGGGACCACCA
GTGGCGAAGGCGTCTTACCAGAACGGGTCCGACGGTGAGGGACGAAAGCTGGGGGCACGAACCGGAT
TAGATACCCGGGTAGTCCCAGCCGTAAACGATGCTCGCTAGGTGTCAGGGACGGTGCGACCGTTTCTGG
TGCCGCAGGGAAGCCGTGAAGCGAGCCACCTGGGAAGTACGGCCGCAAGGCTGAAACTTAAAGGAATT
GGCGGGGGAGCACTACAACGGGTGGAGCCTGCGGTTT

Guaymas_Basin_Arc_ASV0197
CGGTTTGATCAGTCTTCTGGGAAATCTGACAGCTCAACTGTTAGGCTTTCAGGGGATACTGTTAGACTTG
GGACCGGGAGAGGTAAGAGGTACTACAGGGGTAGGAGTGAAATCTTGTAATCCCTGTGGGACCACCA
GTGGCGAAGGCGTCTTACCAGAACGGGTCCGACGGTGAGGGACGAAAGCTGGGGGCACGAACCGGAT
TAGATACCCGGGTAGTCCCAGCCGTAAACGATGTTCGCTAGGTGTCAGGGACGGTGCGACCGTTTCTGG
TGCCGCAGGGAAGCCGTGAAGCGAACCACCTGGGAAGTACGGCCGCAAGGCTGAAACTTAAAGGAATT
GGCGGGGGAGCACTACAACGGGTGGAGCCTGCGGTTT

Guaymas_Basin_Arc_ASV0221
CGGTTTGATCAGTCCTCTGGGAAATCTGACAGCTCAACTGTTAGGCTTCCAGGGGATACTGTCAGACTTG
GGACCGGGAGAGGTAAGAGGTACTACAGGGGTAGGAGTGAAATCTTGTAATCCCTGTGGGACCACCA
GTGGCGAAGGCGTCTTACCAGAACGGGTCCGACGGTGAGGGACGAAAGCTAGGGGCACGAACCGGAT
TAGATACCCGGGTAGTCCTAGCCGTAAACGATGTTCGCTAGGTGTCAGGGACGGTGCGACCGTTTCTGG
TGCCGCAGGGAAGCCGTGAAGCGAACCACCTGGGAAGTACGGCCGCAAGGCTGAAACTTAAAGGAATT
GGCGGGGGAGCACTACAACGGGTGGAGCCTGCGGTTT

Guaymas_Basin_Arc_ASV0465
CGGTTTGATCAGTCTTCTGGGAAATCTGACAGCTCAACTGTTAGGCTTTCAGGGGATACTGTCAGACTTG
GGACCGGGAGAGGTAAGAGGTACTACAGGGGTAGGAGTGAAATCTTGTAATCCCTGTGGGACCACCA
GTGGCGAAGGCGTCTTACCAGAACGGGTCCGACGGTGAGGGACGAAAGCTGGGGGCACGAACCGGAT
TAGATACCCGGGTAGTCCCAGCCGTAAACGATGTTCGCTAGGTGTCAGGGACGGTGCGACCGTTTCTGG
TGCCGCAGGGAAGCCGTGAAGCGAACCACCTGGGAAGTACGGCCGCAAGGCTGAAACTTAAAGGAATT
GGCGGGGGAGCACTACAACGGGTGGAGCCTGCGGTTT

Guaymas_Basin_Arc_ASV0701
CGGTTTGATCAGTCTTCCGGGAAATCTGACAGCTCAACTGTTAGGCTTTCGGGGGATACTGTTAGACTTG
GGACCGGGAGAGGTAAGAGGTACTACAGGGGTAGGAGTGAAATCTTGTAATCCCTGTGGGACCACCA
GTGGCGAAGGCGTCTTACCAGAACGGGTCCGACGGTGAGGGACGAAAGCTGGGGGCACGAACCGGAT
TAGATACCCGGGTAGTCCCAGCCGTAAACGATGTTCGCTAGGTGTCAGGGACGGTGCGACCGTTTCTGG

TGCCGTAGGGAAGCCGTGAAGCGAACCACCTGGGAAGTACGGCCGCAAGGCTGAAACTTAAAGGAATT
GGCGGGGGAGCACTACAACGGGTGGAGCCTGCGGTTT

Guaymas_Basin_Arc_ASV1027
CGGTTTGATCAGTCTTCCGGGAAATCTGACAGCTCAACTGTTAGGCTTTCGGGGGATACTGTTAGACTTG
GGACCGGGAGAGGTAAGAGGTACTACAGGGGTAGGAGTGAAATCTTGTAATCCCTGTGGGACCACCA
GTGGCGAAGGCGTCTTACCAGAACGGGTCCGACGGTGAGGGACGAAAGCTGGGGGCACGAACCGGAT
TAGATACCCGGGTAGTCCCAGCCGTAAACGATGCTCGCTAGGTGTCAGGGACGGTGCGACCGTTTCTGG
TGCCGCAGGGAAGCCGTGAAGCGAGCCACCTGGGAAGTACGGCCGCAAGGCTGAAACTTAAAGGAATT
GGCGGGGGAGCACTACAACGGGTGGAGCCTGCGGTTT
